# Inhibition of TFF3 synergizes with c-MET inhibitors to decrease the CSC-like phenotype and metastatic burden in ER+HER2+ mammary carcinoma

**DOI:** 10.1038/s41419-025-07387-5

**Published:** 2025-02-07

**Authors:** Chuyu He, Xuejuan Wang, Yi-Shiou Chiou, Basappa Basappa, Tao Zhu, Vijay Pandey, Peter E. Lobie

**Affiliations:** 1 Institute of Biopharmaceutical and Health Engineering and Tsinghua Berkeley Shenzhen Institute, Tsinghua Shenzhen International Graduate School, Tsinghua University, Shenzhen, PR China; 2 Master Degree Program in Toxicology, College of Pharmacy, Kaohsiung Medical University, Kaohsiung, Taiwan; 3 Laboratory of Chemical Biology, Department of Studies in Organic Chemistry, University of Mysore, Mysore, India; 4 Shenzhen Bay Laboratory, Shenzhen, Guangdong PR China; 5 Department of Oncology, The First Affiliated Hospital of USTC, Center for Advanced Interdisciplinary Science and Biomedicine of IHM, Division of Life Sciences and Medicine, University of Science and Technology of China, Hefei, Anhui PR China; 6 Hefei National Laboratory for Physical Sciences, University of Science and Technology of China, Hefei, Anhui PR China

**Keywords:** Breast cancer, Targeted therapies

## Abstract

The interaction between HER2 and ERα signaling pathways contributes to resistance to anti-estrogen and HER2-targeted therapies, presenting substantial treatment challenges in ER-positive (ER+) HER2-positive (HER2+) mammary carcinoma (MC). Trefoil Factor-3 (TFF3) has been reported to mediate resistance to both anti-estrogen and anti-HER2 targeted therapies in ER+ and ER+HER2+ MC, respectively. Herein, the function and mechanism of TFF3 in ER+HER2+ MC were delineated; and novel combinatorial therapeutic strategies were identified. Elevated expression of TFF3 promoted the oncogenicity of ER+HER2+ MC cells, including enhanced cell proliferation, survival, anchorage-independent growth, 3D growth, cancer stem cell-like (CSC-like) phenotype, migration, invasion, and xenograft growth. Targeting TFF3 with an interfering RNA plasmid or a small-molecule inhibitor (AMPC) inhibited these oncogenic characteristics, highlighting the therapeutic potential of targeting TFF3 in ER+HER2+ MC. Furthermore, a high-throughput combinatorial anti-cancer compound library screening revealed that AMPC preferentially synergized with receptor tyrosine kinase c-MET inhibitors (c-METis) to reduce cell survival and the CSC-like phenotype. The combination of AMPC and c-METis also synergistically suppressed the in vivo growth of ER+HER2+ MC cell-derived xenografts and abrogated lung metastasis. Mechanistically, TFF3 was observed to activate c-MET signaling through a positive-feedback loop to enhance the CSC-like phenotype of ER+HER2+ MC. Therefore, proof of concept is provided herein that antagonizing of TFF3 is a promising therapeutic strategy in combination with c-MET inhibition for the treatment of ER+HER2+ MC.

## Introduction

Mammary carcinoma (MC) is a heterogeneous disease comprising multiple subtypes with unique biological characteristics that influence responses to treatment modalities and clinical outcomes [1]. Human epidermal growth factor receptor 2 (HER2) gene amplification occurs in 13–22% of MC cases, characterized by increased proliferation, tumor growth and metastatic potential [2, 3], contributing to aggressive behavior with an unfavorable clinical prognosis. Approximately 60–70% of HER2+ MC cases also express estrogen receptor-α (ERα) [3]. This molecular characteristic distinguishes the ER+HER2+ MC subtype, which accounts for approximately 10% of MC cases, as a distinct subtype [4]. Research has demonstrated that administration of combined anti-estrogen and HER2-targeted therapy to ER+HER2+ MC patients significantly improved overall response rate, clinical benefit rate, and progression-free survival compared to anti-estrogen monotherapy [5]. However, reports have identified a complex bidirectional crosstalk between the ER and HER2 pathways, an intrinsic interaction that impedes therapeutic effectiveness [3, 4]. Furthermore, current treatment approaches for ER+HER2+ MC have limited therapeutic efficacy due to the constraints of anti-estrogen and HER2-targeted therapies and lack of predictive markers for therapeutic resistance [4, 6]. These challenges in ER+HER2+ MC necessitate the exploration of targeted therapies that utilize novel regulatory mechanisms.

Trefoil factor 3 (TFF3) is a 59 amino acid peptide initially observed to be secreted by intestinal goblet cells and reported to effect mucosal repair in the gastrointestinal tract [7]. Recent findings have suggested that the biological role of TFF3 extends beyond mucosal protection, and is also associated with oncogenic progression in different types of cancer [7]. TFF3 expression is elevated and often associated with prognosis in gastric [8], colorectal [9], hepatocellular [10], thyroid [11], lung [12], pancreatic [13], prostate [14], cervical [15], endometrial [16], and ER+ mammary carcinomas [17–21]. Functionally, TFF3 has been reported to interact with CXCR4/7 [22], CD147 [23], CRP-ductin/DMBT1^gp340^ [24], PAR2 [25], and LINGO2 [26]. In addition, TFF3 enhances the activation of HER1-4 [18], c-MET [18], IGFR1 [18], and c-SRC [18, 19], and increases the expression of multiple WNT ligands [13], thereby promoting downstream signaling pathways including p44/42 MAPK [9], NF-κB [27], PI3K/AKT [28, 29] STAT3 [15, 16], and WNT [13] to promote cancer cell survival, proliferation, a cancer stem cell-like (CSC-like) phenotype, invasion, migration and metastasis [7, 30]. In ER+HER2+ MC, a bidirectional crosstalk between ER and HER2 contributes to resistance against both anti-estrogens and HER2-targeted therapies [3, 31], with TFF3 implicated in this resistance mechanism [17, 18, 20, 32]. As an estrogen-responsive gene [32], TFF3 is positively correlated with ER+ status (at least in Caucasian MC patient cohorts), and its high expression is associated with a poor prognosis in ER+ MC patients following endocrine therapy [17, 32–34]. Furthermore, TFF3 has been demonstrated to enhance ERα transcriptional activity in MC, leading to the stimulation of estrogen-independent proliferation and reduced responsiveness to anti-estrogens [17, 20, 32]. Specifically, TFF3 is elevated in MC resistant to tamoxifen [17, 20] and aromatase inhibition [35]. Conversely, TFF3 depletion or inhibition resensitizes resistant cells to the respective anti-estrogen treatment [17, 20, 35]. As indicated above, a previous study has suggested bidirectional functional cross-regulation between HER2 and TFF3, partially independent of ERα in ER+HER2+ MC [18]. Specifically, TFF3 expression is decreased by HER2 activation and increased upon trastuzumab inhibition of HER2; and TFF3 is functionally involved in mediating acquired trastuzumab resistance in ER+HER2+ MC [18]. Given that the dysregulation of ER and HER pathways in MC can be the result of the activation of downstream signaling pathways including p44/42 MAPK and PI3K/AKT [36] which are promoted by TFF3, the reported high TFF3 expression [19] and its role in ER and HER2 signaling [17, 18, 20, 32], targeting TFF3 offers a potential therapeutic approach for ER+HER2+ MC to overcome the resistance of anti-estrogens and/or HER2-targeted therapies and improve clinical outcomes for patients.

As a transmembrane receptor tyrosine kinase (RTK), c-MET has been identified as a receptor for hepatocyte growth factor (HGF) and documented to be increased in expression and activity in MC [37, 38]. c-MET amplification plays a crucial role in MC mediating cellular responses including proliferation, migration, and invasion [38], demonstrating a function in maintaining an oncogenic phenotype. The co-expression and activation of c-MET with other RTKs hold prognostic importance in MC, lung carcinoma, and glioma [39]. Furthermore, the interplay between c-MET and other RTKs significantly impacts resistance to anti-RTK treatments, which is a critical concern in MC therapy [38, 40].

Considering that targeted therapies often face challenges such as the development of resistance, combinations that target complimentary pathways or potential escape mechanisms appear to be more effective than single or sequential therapy [41]. Herein, novel combinatorial therapy regemins were investigated to identify potential strategies that may improve treatment outcomes in ER+HER2+ MC. High-throughput screening of 247 anticancer compounds in combination with a small molecule inhibitor of TFF3 (named AMPC) was performed in MDA-MB-361 and BT474 cells. The results demonstrated that targeting TFF3 not only enhances the sensitivity of cells to protein tyrosine kinase inhibitors but also prevents the feedback mechanisms consequent to utilization of these inhibitors, thereby achieving superior synergistic efficacy. Among these agents, c-MET inhibitors exhibited high synergy with TFF3 inhibition. The synergistic actions of the drug combinations in the treatment of ER+HER2+ MC in vitro and in vivo, using orthotopic ER+HER2+ MC xenograft models were demonstrated. These results indicated that combining TFF3 and c-MET inhibition may serve as a novel therapeutic strategy for ER+HER2+ MC patients.

## Methods

### Cell culture and transfection

MDA-MB-361 and BT474 cell lines were obtained from BeNa Culture Collection Co. Ltd (Suzhou, China) and Procell Life Science & Technology Co. Ltd (Wuhan, China), respectively. MDA-MB-361 cells were cultured at 37 °C in a CO_2_-free incubator with L15 medium, supplemented with 10% fetal bovine serum (FBS) and 1% penicillin–streptomycin (P/S) (Thermo Fisher Scientific, Waltham, MA, USA). BT474 cells were maintained at 37 °C in a 5% CO_2_ incubator with RPMI-1640 medium, supplemented with 20% FBS, 1% P/S, 2 mM L-glutamine, and 10 ng/mL insulin. All experiments were performed in the respective media containing 2% FBS.

As previous studies reported [17], MDA-MB-361 and BT474 cells underwent stable transfection with the *pIRESneo3-TFF3 plasmid* to force the expression of TFF3, facilitated by Lipofectamine 3000 reagent (Thermo Fisher Scientific, Waltham, MA, USA). Simultaneously, cells were transfected with the *pIRESneo3-vector plasmid* as control. G418 selection was applied at a concentration of 400 µg/mL for 4 weeks. Moreover, MDA-MB-361 cells underwent transfection with a *shRNA plasmid* targeting TFF3 to generate stable TFF3-depleted cells, employing a scrambled *shRNA plasmid* for comparison. Hygromycin B selection was utilized at a concentration of 200 µg/mL for 4 weeks [18]. These established cell lines are denoted as MDA-MB-361-TFF3, MDA-MB-361-VEC, BT474-TFF3, BT474-VEC, MDA-MB-361-shTFF3, and MDA-MB-361-shCtrl cells. For transient RNA interference, MDA-MB-361 and BT474 cells were transfected with plasmids containing scrambled siRNA, siMET #1, or siMET #2 sourced from GENEWIZ (Suzhou, China). The specifications for siMET #1 and siMET #2 employed in the study were as follows,

siMET #15’-AGAAUGUCAUCAUUCAGGCTT-3’

siMET #25’-UACUCAGCAACCUUCUGAAGGTT-3’

### Reagents

The 247 anti-cancer compounds library (Cambridge Cancer Compound Library, Catalog No. L2300) utilized in this study was acquired from SelleckChem (Houston, TX, USA). Additionally, three c-MET inhibitors, namely Cabozantinib, SU11274, and PHA-665752 were sourced from SelleckChem (Houston, TX, USA).

### Western blot (WB) analysis

Radio Immunoprecipitation Assay Lysis buffer was employed for cell lysis, followed by sodium dodecyl sulfate (SDS) polyacrylamide gel electrophoresis (PAGE) to fractionate the proteins within the cell lysate. WB analysis was carried out following established protocols [42]. Immunoblots were developed using a Gel Imager System (Bio-Rad, USA). Details of the antibodies utilized are provided in Supplementary information 11.

### Cell function assays and enzyme-linked immunosorbent assay (ELISA)

Monolayer cell viability, Annexin-V/Propidium Iodide staining, CASPASE 3/7 activity, foci formation, 3D-Matrigel growth, and spheroid formation assays were conducted according to established protocols [43, 44]. For monolayer cell viability assay, 3000 cells were seeded in 2% serum containing medium in 96-well plate and incubated for 6 days, using the AlamarBlue assay reagent (BioChip, Beijing, China). To analyze apoptotic cell death, cells were seeded at 50–70% confluency in a 6-cm dish and allowed to adhere overnight before treatment. After a 3-day drug treatment period, the cells were harvested and resuspended in 400 μL of Annexin V binding buffer per sample. Subsequently, 5 μL of Annexin V-Alexa Fluor 488 staining solution was added, followed by a 15-min incubation period in the absence of light. 5 μL of 7-AAD staining solution was then introduced, followed by a subsequent 5-minute incubation. Samples were analyzed within 30 min using a Cytoflex Flow Cytometry system (Beckman Coulter, CA, USA). For CASPASE 3/7 activity assay, cells were seeded at a density of 2 × 10^4^ cells per well in a 96-well plate and evaluated 3 days post-seeding or drug treatment using the Caspase-Glo^®^ 3/7 Assay kit (Promega, Madison, WI, USA) according to the manufacturer’s instructions. For foci formation assay, 3000 cells were plated into 24-well plates in medium supplemented with 2% FBS at 37 °C for 2 weeks until foci formation. Then, the foci were fixed with formalin and stained with crystal violet (Sigma-Aldrich, MO, USA). Survival fraction was measured by eluting the crystal violet with methanol and absorbance was detected at 595 nm. 3D-Matrigel assays were performed in 48-well plates coated with Matrigel (Corning, MA, USA) and allowed to solidify for 30 min. 2000 cells were then plated to the pre-coated plates in the medium supplemented with 2% FBS and 4% Matrigel for 12 days. The medium was refreshed every 3 days. AlamarBlue (BioChip, Beijing, China) was used to determine cell viability at the end of the experiment. In spheroid formation assays, 1000 cells were seeded in a 24-well ultralow attachment plates (Corning, MA, USA) culturing with serum-free medium supplemented with P/S, 10 ng/mL recombinant human basic FGF, 20 ng/mL recombinant human EGF, 2% B27, and 5 μg/mL bovine insulin. Spheroids with diameter exceeding 50 μm were counted under a microscope (Olypus, Tokyo, Japan) after 12 days. The concentration of secreted TFF3 from cells and serum TFF3 levels were determined using the Quantikine^®^ ELISA Human TFF3 Immunoassay kit (R&D Systems, Minneapolis, MN, USA) following the manufacturer’s protocol. Cells were plated at a density of 3 × 10^5^ cells in a 6 cm dish and cultured with 600 μL of serum-free medium for 2 days. Subsequently, the supernatant was collected as the sample. Furthermore, a fresh blood sample was obtained from the cardiac puncture of euthanized mice and subjected to centrifugation at 4 °C for 10 min at 3000 rpm to isolate the serum, which was then stored at −80 °C for subsequent analyses.

### ALDEFLUOR assay

The ALDEFLUOR assay was performed using the ALDEFLUOR assay kit (STEMCELL Technologies, Vancouver, Canada) in accordance with the manufacturer’s instructions. Cells were seeded at 50–70% confluency in 6-well plate and were exposed to the respective drugs for 3 days. The ALDEFLOUR activity was assessed via fluorescence-activated cell sorting analysis. Re-suspended cells were exposed to the ALDEFLUOR substrate (BAAA, BODIPY®-aminoacetaldehyde) to identify the ALDH1-positive population, and the baseline fluorescence was established using a specific ALDH1 inhibitor, diethylaminobenzaldehyde (DEAB).

### Real-time quantitative polymerase chain reaction (qPCR) analysis

Tissue samples were first rinsed with sterile saline and subsequently lysed in TRIzol (Sigma-Aldrich, MO, USA) for subsequent RNA extraction. Total RNA extraction followed established protocols, which included DNase I treatment, conversion of total RNA to complementary DNA (cDNA), PCR, and qPCR assays, conducted as previously described [45]. The qPCR procedure was carried out according to the outlined methodology [45]. The primers employed for qPCR were as follows,

*hHPRT1* forward: 5**′**-TTCCTTGGTCAGGCAGTATAATCC-3**′**

*hHPRT1* reverse: 5**′**-AGTCTGGCTTATATCCAACACTTCG-3**′**

*mgapdh* forward: 5**′**-CTCACTCAAGATTGTCAGCAATG-3**′**

*mgapdh* reverse: 5**′**-CACATTGGGGGTAGGAACAC-3**′**

### Co-immunoprecipitation (Co-IP) assay

Whole-cell lysates were obtained by extracting cells with a cell lysis buffer containing protease inhibitor cocktail (TargetMol, Shanghai, China). Subsequently, the normalized lysates were incubated overnight at 4 °C with gentle agitation after adding 5 µg of primary or anti-IgG antibodies. The lysate–antibody mixture was then incubated with magnetic protein G DynabeadsTM (Thermo Fisher Scientific, Waltham, MA, USA) and washed thrice with protein binding buffer (composed of 150 mM NaCl, 20 mM Tris pH 8.0, 1% NP-40, and supplemented with protease and phosphatase inhibitors). The immunoprecipitant was eluted in sample buffer containing 1% β-mercaptoethanol and subjected to SDS-PAGE immunoblotting. Details of the antibodies used are listed in Supplementary information 11.

### Xenografts

All animal experiments were conducted with the approval of the Institutional Animal Care and Use Committee of the Laboratory Animal Centre of Peking University Shenzhen Graduate School (Certificate number: YW) and the “Ethical Development no. 9 (year 2020)” from Tsinghua Shenzhen International Graduate School as previously described [20]. Specific pathogen-free (SPF) female BALB/c athymic nude mice were obtained from Guangdong Vital River Laboratory Animal Technology Co. (Foshan, China) and housed in an SPF animal facility with ad libitum access to clean water and food. After a 1-week acclimation period, eight mice were randomly assigned to each subgroup. Mice were subcutaneously implanted with 0.72 mg 90-day release 17β-estradiol pellets (Innovative Research of America, Sarasota, FL, USA) at the back of the neck. Following a 3-day interval, 1 × 10^7^ MDA-MB-361 cells were orthotopically implanted into the right fourth mammary fat pad of mice to establish a xenograft model. Animal weight and xenograft volume were monitored daily. The xenograft volume was calculated using the formula 0.52 × (length × width^2^) [19]. Once the xenograft volumes reached ~80–100 mm³, the xenograft-bearing mice were treated daily for a 2-week period. The treatments included intraperitoneal administration of either the vehicle (1% DMSO/10% PEG400 in distilled saline) or 20 mg/kg AMPC, or intragastric administration of 60 mg/kg Cabozantinib (SelleckChem, Houston, TX, USA). The mice were sacrificed after a two-week treatment period.

### Immunohistochemistry (IHC) staining and TUNEL assays

IHC staining was conducted by employing the labeled streptavidin-biotin-peroxidase complex method [13]. TUNEL assay was performed by using TUNEL Assay Kit (Abcam, Waltham, MA, USA), as previously described [13, 21]. Subsequent scoring of IHC staining was performed utilizing the immunoreactive score (IRS) method [46]. Two independent researchers were blinded and meticulously assessed and validated the staining results. Details regarding the antibodies employed are provided in Supplementary information 11.

### Statistical analysis

Graphical representations and statistical analyses were performed utilizing GraphPad Prism 9 (GraphPad Software, Inc., CA, USA). The statistical significance between two groups was assessed using a two-tailed unpaired Student’s *t*-test, while analysis of variance (ANOVA) was employed for comparisons among multiple treatment groups. Statistical significance thresholds were set at *P < 0.05, **P < 0.01, and ***P < 0.001. Data conforming to a normal distribution were presented as mean ± standard deviation (SD).

## Results

### Forced expression of TFF3 enhances oncogenic behavior in ER+HER2+ MC cells

**Fig. 1:**
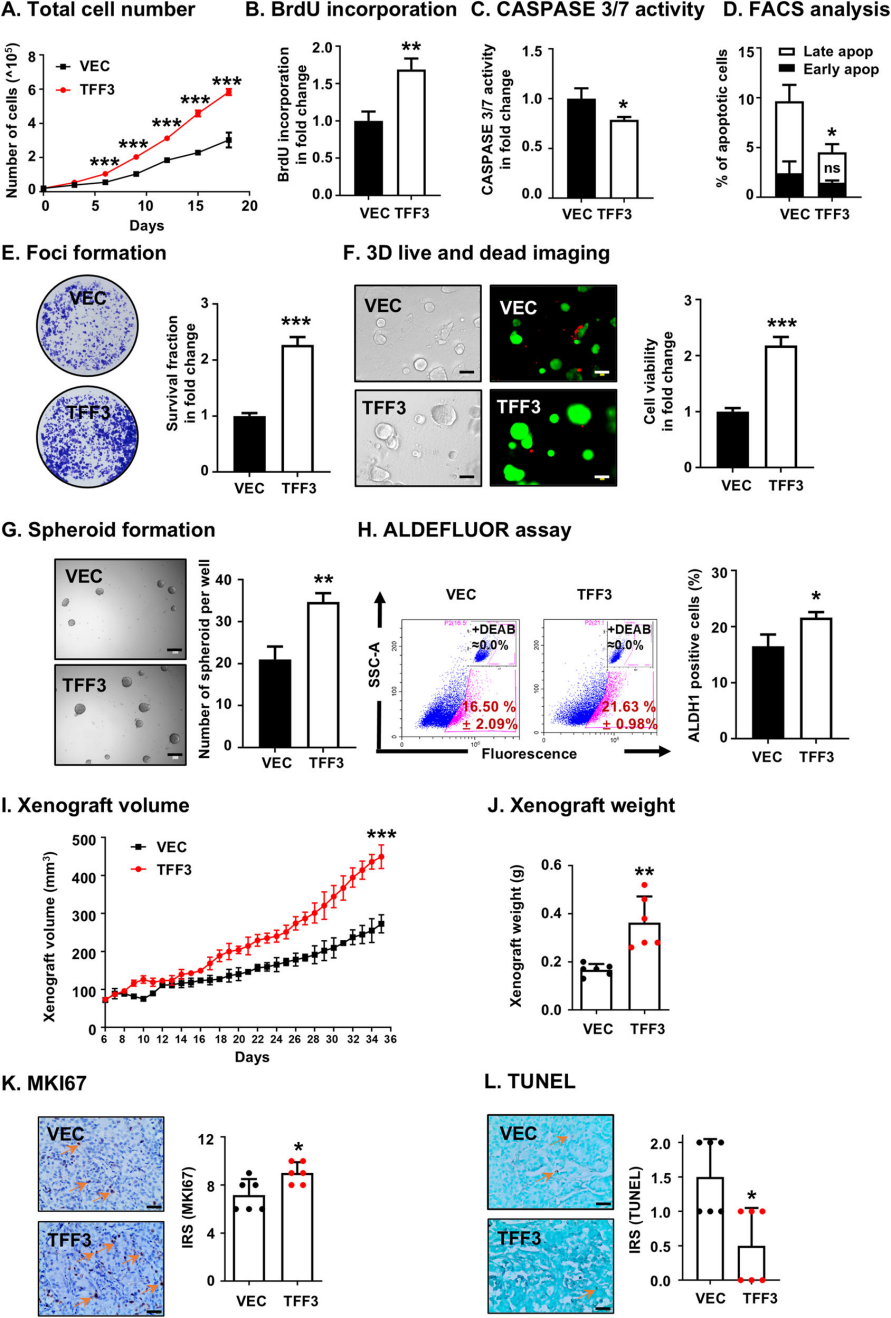
Forced expression of TFF3 enhances oncogenic behaviors in MDA-MB-361 cells. MDA-MB-361 cells were stably transfected with *pIRESneo3-TFF3* (designated MDA-MB-361-TFF3/TFF3) or *pIRESneo3-vector* (MDA-MB-361-VEC/VEC) plasmid. **A** Total cell number counting. Data are expressed as mean ± SD (*n* = 3). Statistical significance is indicated as *P < 0.05, **P < 0.01, and ***P < 0.001. **B** BrdU incorporation assay was performed after 12 h of serum deprivation. Data are expressed as mean ± SD (*n* = 3). Statistical significance is indicated as *P < 0.05, **P < 0.01, and ***P < 0.001. **C** CASPASE 3/7 activity was assessed after 12 h of serum deprivation. Data are expressed as mean ± SD (*n* = 3). Statistical significance is indicated as *P < 0.05, **P < 0.01, and ***P < 0.001. **D** Apoptotic cell death was determined after 12 h of serum deprivation by Annexin-V/PI. The percentages of cells in early or late apoptotic phase were analyzed. Data are expressed as mean ± SD (*n* = 3). Statistical significance is indicated as *P < 0.05, **P < 0.01, and ***P < 0.001. **E** Foci formation was assessed with MDA-MB-361-VEC and MDA-MB-361-TFF3 cells cultured in monolayer at low cell density for 14 days. Foci were stained by crystal violet and fold change in survival fraction was measured by eluting the crystal violet with methanol and detecting absorbance at 595 nm. Data are expressed as mean ± SD (*n* = 3). Statistical significance is indicated as *P < 0.05, **P < 0.01, and ***P < 0.001. **F** Microscopic visualization of LIVE/DEAD^TM^ Cell Imaging kit-stained colonies was conducted after 12 days of cell culturing in medium containing 2% FBS and 4% Matrigel. The bright-field images are displayed on the left, while the merged images of live and dead cells are on the right, with red indicating dead colonies and green indicating live colonies. Scale bars, 100 μm. Fold change in cell viability of 3D colonies was determined by AlamarBlue (BioChip, Beijing, China). Data are expressed as mean ± SD (*n* = 3). Statistical significance is indicated as *P < 0.05, **P < 0.01, and ***P < 0.001. **G** Spheroid formation assay was performed with MDA-MB-361-VEC and MDA-MB-361-TFF3 cells seeded in ultralow attachment plates and cultured in spheroid growth media for 12 days. Scale bar, 100 μm. The spheroids with diameters greater than 50 μm in each well were counted. Data are expressed as mean ± SD (*n* = 3). Statistical significance is indicated as *P < 0.05, **P < 0.01, and ***P < 0.001. **H** ALDEFLUOR activity was measured after 12 h of serum deprivation. The cells were harvested and incubated with ALDEFLUOR substrate to define the ALDH1-positive population. DEAB was used to establish the baseline fluorescence. The percentage of ALDH1-positive cells (in the pink box) was plotted by flow cytometry. Data are expressed as mean ± SD (*n* = 3). Statistical significance is indicated as *P < 0.05, **P < 0.01, and ***P < 0.001. **I** In vivo xenograft growth was evaluated by xenograft volumes (mm^3^) every day until the 35th day. Significance analysis was performed on xenograft volumes at the conclusion of the experiment (Day 35). Data are expressed as mean ± SD (*n* = 6). Statistical significance is indicated as *P < 0.05, **P < 0.01, and ***P < 0.001. **J** Xenograft weight of each group of animals after sacrifice. Data are expressed as mean ± SD (*n* = 6). Statistical significance is indicated as *P < 0.05, **P < 0.01, and ***P < 0.001. **K** Representative micrographs and IRS of IHC staining for MKI67 in the indicated xenografts. Scale bar, 20 μm. Data are expressed as mean ± SD (*n* = 6). Statistical significance is indicated as *P < 0.05, **P < 0.01, and ***P < 0.001. **L** Representative micrographs and IRS of TUNEL in the indicated xenografts. Scale bar, 20 μm. Data are expressed as mean ± SD (*n* = 6). Statistical significance is indicated as *P < 0.05, **P < 0.01, and ***P < 0.001.

To elucidate the functions of TFF3 in ER+HER2+ MC, MDA-MB-361, and BT474 cells stably transfected with TFF3 cDNA were generated and validated through western blot analysis (Supplementary information 1A, 2A). Both MDA-MB-361 and BT474 cells with forced expression of TFF3 demonstrated a significant increase in total cell number compared to the respective vector-transfected counterparts (Fig. 1A and Supplementary information 2B). In addition, MDA-MB-361-TFF3 and BT474-TFF3 cells demonstrate an increased propensity for S-phase entry, as compared to their respective vector-transfected cells as determined by the BrdU incorporation assay (Fig. 1B and Supplementary information 2C), indicating that TFF3 stimulated ER+HER2+ MC cell cycle progression and proliferation. Additionally, ER+HER2+ MC cells stably transfected with TFF3 demonstrated a significant decrease in CASPASE 3/7 activity (Fig. 1C and Supplementary information 2D) and reduced apoptotic cell death, particularly in the late stage of apoptosis (Fig. 1D and Supplementary information 1B, 2E), compared to their respective vector transfected counterparts.

Loss of contact inhibition and anchorage-independent growth is a key hallmark of oncogenic transformation and cancer progression [47]. In order to examine and assess this characteristic of cancer cells in vitro, foci formation assays were performed. MDA-MB-361-TFF3 and BT474-TFF3 cells exhibited enhanced anchorage-independent growth as compared to MDA-MB-361-VEC and BT474-VEC cells (Fig. 1E and Supplementary information 2F). In 3D-Matrigel culture, which more closely mimics in vivo conditions, ER+HER2+ MC cells with forced expression of TFF3 demonstrated higher viability, characterized by increased number of live cells and reduced number of dead cells compared to their vector-transfected counterparts (Fig. 1F and Supplementary information 2G). These results demonstrated that TFF3 promotes proliferation, cell survival and anchorage-independent growth in ER+HER2+ MC cells in vitro and promotes cancer cell colony growth in 3D matrigel.

Cancer cell migration and invasion are pivotal for cancer progression, and directly contribute to metastatic dissemination [47]. Transwell assays were conducted to assess the effect of forced expression of TFF3 on the migratory and invasive capacities of ER+HER2+ MC cells. The results demonstrated that forced expression of TFF3 in MDA-MB-361 cells led to increased migration and invasion compared to vector-transfected cells (Supplementary information 1C, D). Similarly, in BT474 cells, forced expression of TFF3 resulted in increased migration and invasion (Supplementary information 2H, I).

CSCs play a crucial role in MC progression, possessing the ability for self-renewal and tumor-initiating capacity [48]. Prior studies have reported elevated TFF3 expression in breast cancer stem cells (BCSCs) [18, 20]. Herein, the effect of TFF3 on the CSC-like phenotype of ER+HER2+ MC cells was assessed. Spheroid formation assays revealed that the number of spheroids in MDA-MB-361 and BT474 cells with forced expression of TFF3 were higher in comparison to their respective vector-transfected counterparts (Fig. 1G and Supplementary information 2J). Consistent results were observed by ALDEFLUOR assay, which demonstrated that MDA-MB-361 and BT474 cells with forced expression of TFF3 exhibited a significantly higher population of ALDH1-positive cells compared to the vector-transfected control (Fig. 1H and Supplementary information 2K). Hence, TFF3 promotes the CSC-like phenotype in ER+HER2+ MC cells.

Subsequently, the impact of TFF3 on ER+HER2+ MC growth in vivo was assessed in a MDA-MB-361 xenograft model. Consistent with the in vitro findings, MDA-MB-361 xenografts generated by cells with forced expression of TFF3 exhibited significant increases in both volume and weight compared to vector-transfected cells at the end of the experiment (Fig. 1I, J). Further analysis of xenograft specimens by IHC determined the effect of TFF3 on xenograft cell proliferation and apoptosis in vivo using MKI67 staining and TUNEL assay respectively. The higher proportion of MKI67-positive cells in MDA-MB-361-TFF3 cell-derived xenografts indicated increased proliferation (Fig. 1K). The xenografts generated from MDA-MB-361-TFF3 cells also contained significantly fewer apoptotic nuclei than MDA-MB-361 formed from MDA-MB-361-vector cells in the TUNEL assay (Fig. 1L). These results indicate that forced expression of TFF3 promotes in vivo growth of MDA-MB-361 cells. Hence, the forced expression of TFF3 augmented the oncogenicity of ER+HER2+ MC cells in vitro and in vivo.

### Depletion of TFF3 suppresses oncogenic behaviors in ER+HER2+ MC cells

**Fig. 2:**
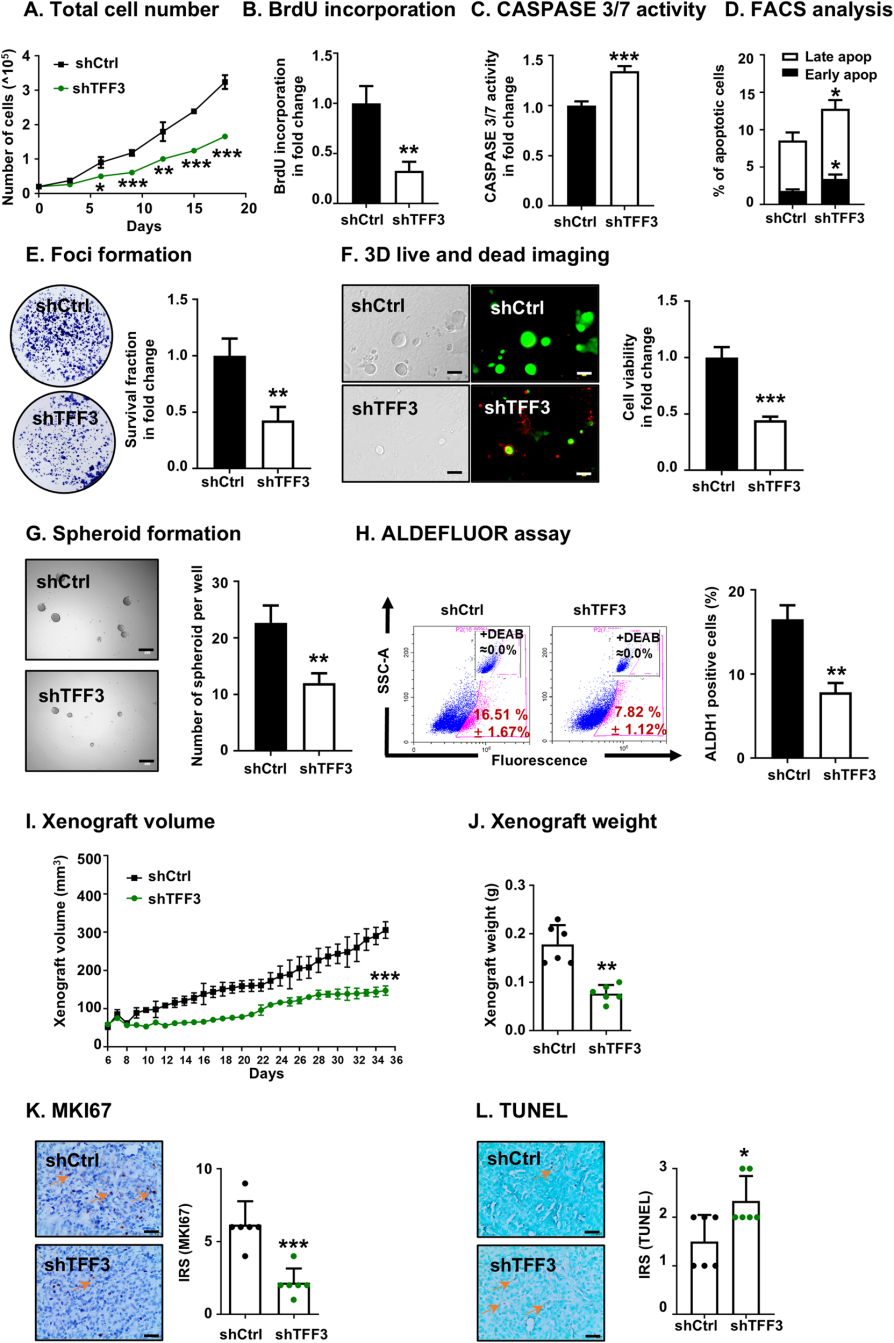
Depletion of TFF3 suppresses oncogenic behaviors in MDA-MB-361 cells. MDA-MB-361 cells were stably transfected with *pSilencersiTFF3* (designated MDA-MB-361-shTFF3/shTFF3) or empty vector (MDA-MB-361-shCtrl/shCtrl) plasmid. **A** Total cell number counting. Data are expressed as mean ± SD (*n* = 3). Statistical significance is indicated as *P < 0.05, **P < 0.01, and ***P < 0.001. **B** BrdU incorporation assay was performed after 12 h of serum deprivation. Data are expressed as mean ± SD (*n* = 3). Statistical significance is indicated as *P < 0.05, **P < 0.01, and ***P < 0.001. **C** CASPASE 3/7 activity was assessed after 12 h of serum deprivation. Data are expressed as mean ± SD (*n* = 3). Statistical significance is indicated as *P < 0.05, **P < 0.01, and ***P < 0.001. **D** Apoptotic cell death was determined after 12 h of serum deprivation by Annexin-V/PI. The percentages of cells in early or late apoptotic phase were analyzed. Data are expressed as mean ± SD (*n* = 3). Statistical significance is indicated as *P < 0.05, **P < 0.01, and ***P < 0.001. **E** Foci formation was assessed with MDA-MB-361-shCtrl and MDA-MB-361-shTFF3 cells cultured in monolayer at low cell density for 14 days. Foci were stained by crystal violet, and fold change in survival fraction was measured by absorbance at 595 nm. Data are expressed as mean ± SD (*n* = 3). Statistical significance is indicated as *P < 0.05, **P < 0.01, and ***P < 0.001. **F** Microscopic visualization of LIVE/DEAD^TM^ Cell Imaging kit-stained colonies was conducted after 12 days of cell culturing in medium containing 2% FBS and 4% Matrigel. The bright-field images are displayed on the left, while the merged images of live and dead cells are on the right, with red indicating dead colonies and green indicating live colonies. Scale bars, 100 μm. fold change in cell viability of 3D colonies was determined by AlamarBlue (BioChip, Beijing, China). Data are expressed as mean ± SD (*n* = 3). Statistical significance is indicated as *P < 0.05, **P < 0.01, and ***P < 0.001. **G** Spheroid formation assay was performed with MDA-MB-361-VEC and MDA-MB-361-TFF3 cells seeded in ultralow attachment plates and cultured in spheroid growth media for 12 days. Scale bar, 100 μm. The spheroids with diameters greater than 50 μm in each well were counted. Data are expressed as mean ± SD (*n* = 3). Statistical significance is indicated as *P < 0.05, **P < 0.01, and ***P < 0.001. **H** ALDEFLUOR activity was measured after 12 h of serum deprivation. The cells were then harvested and incubated with ALDEFLUOR substrate to define the ALDH1-positive population. DEAB was used to establish the baseline fluorescence. The percentage of ALDH1-positive cells (in the pink box) was plotted by flow cytometry. Data are expressed as mean ± SD (*n* = 3). Statistical significance is indicated as *P < 0.05, **P < 0.01, and ***P < 0.001. **I** In vivo xenograft growth was evaluated by xenograft volumes (mm^3^) every day until the 35th day. Significance analysis was performed on xenograft volumes at the conclusion of the experiment (Day 35). Data are expressed as mean ± SD (*n* = 6). Statistical significance is indicated as *P < 0.05, **P < 0.01, and ***P < 0.001. **J** Xenograft weight of each group of animals after sacrifice. Data are expressed as mean ± SD (*n* = 6). Statistical significance is indicated as *P < 0.05, **P < 0.01, and ***P < 0.001. **K**. Representative micrographs and IRS of IHC staining for MKI67 in the indicated xenografts. Scale bar, 20 μm. Data are expressed as mean ± SD (*n* = 6). Statistical significance is indicated as *P < 0.05, **P < 0.01, and ***P < 0.001. **L** Representative micrographs and IRS of TUNEL in the indicated xenografts. Scale bar, 20 μm. Data are expressed as mean ± SD (*n* = 6). Statistical significance is indicated as *P < 0.05, **P < 0.01, and ***P < 0.001.

A MDA-MB-361 cell model with depleted TFF3 expression was generated by a shRNA-based approach (designated as MDA-MB-361-shTFF3) and validated through western blot analysis (Supplementary information 3A). Resulting functional consequences were investigated by using approaches similar to those used to examine the consequences of forced expression of TFF3 (Fig. 1, Supplementary information 1-2). TFF3-depleted MDA-MB-361 cells exhibited a significant decrease in total cell number (Fig. 2A) and BrdU incorporation (Fig. 2B). Additionally, depleted expression of TFF3 significantly increased CASPASE 3/7 activity and promoted apoptosis (Fig. 2C, D and Supplementary information 3B). TFF3-depleted MDA-MB-361 cells demonstrated markedly reduced foci formation (Fig. 2E). Similarly, in 3D-Matrigel culture, MDA-MB-361-shTFF3 cells formed fewer colonies and demonstrated reduced viability, characterized by a higher number of dead cells and fewer live cells as compared to the vector control cells (Fig. 2F). The depletion of TFF3 significantly reduced migration and invasion of MDA-MB-361 cells (Supplementary information 3C, D). Additionally, spheroid formation assays indicated that TFF3-depleted MDA-MB-361 cells formed significantly fewer spheroids compared to the control vector cells (Fig. 2G). Consistently, ALDEFLUOR assay results revealed a significantly lower population of ALDH1-positive cells in TFF3-depleted MDA-MB-361 cells (Fig. 2H).

The in vivo growth of TFF3-depleted MDA-MB-361 cells was also evaluated. The results demonstrated that TFF3 depleted MDA-MB-361 cell-generated xenografts exhibited significantly reduced growth in volume compared to the control vector xenografts, a finding further confirmed by the lower xenograft weights at the end of the experiment (Fig. 2I, J). IHC and TUNEL analysis additionally revealed a decreased proportion of MKI67-positive cells, and higher apoptosis in the TFF3-depleted xenografts compared to the control vector cell generated xenografts (Fig. 2K, L). Thus, the depletion of TFF3 decreased oncogenicity of ER+HER2+ MC cells both in vitro and in vivo.

### AMPC inhibited the oncogenicity of ER+HER2+ MC cells

TFF3 has been reported to form homodimers via the seventh cysteine (Cys57) residue, and the homodimeric form of TFF3 plays a critical role in inhibiting apoptosis [7, 30]. Recently, a small-molecule inhibitor named AMPC (*2-amino-4-(4-(6-fluoro-5-methylpyridin-3-yl)phenyl)-5-oxo-4H,5H-pyrano [3,2-c]chromene-3-carbonitrile*) was developed that interfered with dimerization of TFF3 via the Cys57 residue [30]. AMPC leads to the rapid degradation of the monomeric form of TFF3, thereby diminishing TFF3-mediated signaling pathways crucial for cancer cell survival [9, 12, 13, 20, 30]. Given the observed oncogenic functions of TFF3 in ER+HER2+ MC cells, AMPC was employed to investigate the effect and therapeutic potential of small molecule-mediated inhibition of TFF3 in ER+HER2+ MC cells.

**Fig. 3:**
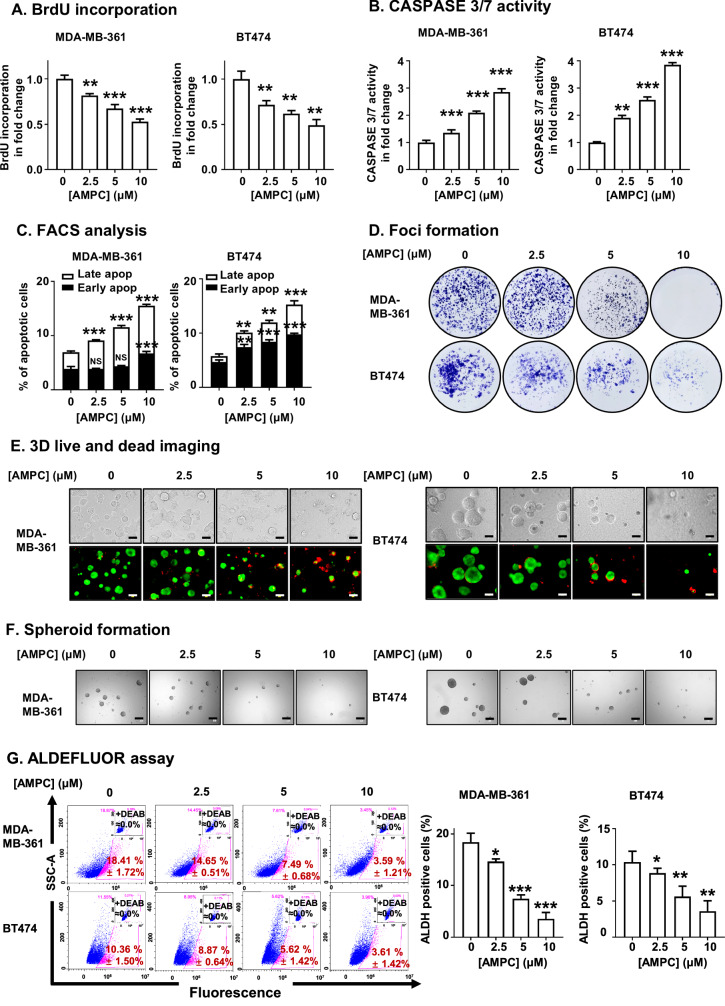
AMPC inhibits the oncogenicity of ER+HER2+ MC cells*.* **A** BrdU incorporation assay in MDA-MB-361 and BT474 cells treated with 0, 2.5, 5, or 10 µM AMPC for 3 days. Data are expressed as mean ± SD (*n* = 3). Statistical significance is indicated as *P < 0.05, **P < 0.01, and ***P < 0.001. **B** CASPASE 3/7 activities were determined after 3 days of AMPC (0, 2.5, 5, or 10 µM) treatment. Data are expressed as mean ± SD (*n* = 3). Statistical significance is indicated as *P < 0.05, **P < 0.01, and ***P < 0.001. **C** Apoptotic cell death of MDA-MB-361 and BT474 cells was determined after 2 days of AMPC treatment (0, 2.5, 5, or 10 µM) by Annexin-V/PI. The percentages of early apoptotic and late apoptotic are shown. Data are expressed as mean ± SD (*n* = 3). Statistical significance is indicated as *P < 0.05, **P < 0.01, and ***P < 0.001. **D** Foci formation was conducted with MDA-MB-361 and BT474 cells treated with 0, 2.5, 5, or 10 µM AMPC in monolayer culture at low cell density for 14 days. Foci were stained by crystal violet. **E** Microscopic visualization of colonies stained with the LIVE/DEAD™ Cell Imaging Kit after 12 days of AMPC treatment (0, 2.5, 5, or 10 µM) of MDA-MB-361 and BT474 cells cultured in medium containing 2% FBS and 4% Matrigel after 3 days of pre-culture. The bright-field images are shown above, and the merged images of live (green) and dead (red) colonies are shown below. Scale bars, 100 μm. **F** Spheroid formation assay. MDA-MB-361 and BT474 cells were seeded in ultralow attachment plates and cultured in spheroid growth media for 12 days with AMPC treatment (0, 2.5, 5, or 10 µM). Scale bar, 100 μm. **G** ALDEFLUOR activity was measured after 3 days of AMPC treatment (0, 2.5, 5, or 10 µM). MDA-MB-361 and BT474 cells were then harvested and incubated with ALDEFLUOR substrate to define the ALDH1-positive population. DEAB was used to establish the baseline fluorescence. The percentage of ALDH1-positive cells (in the pink box) was plotted by flow cytometry. Data are expressed as mean ± SD (*n* = 3). Statistical significance is indicated as *P < 0.05, **P < 0.01, and ***P < 0.001.

The IC_50_ value of AMPC in MDA-MB-361 (3.206 ± 0.757 µM) and BT474 cells (2.268 ± 0.605 µM) was determined using total cell number assays (Supplementary information 4A). Consistent with the effect of shRNA-mediated depletion of TFF3, inhibition of TFF3 by AMPC decreased the BrdU incorporation of MDA-MB-361 and BT474 cells, and in a dose-dependent manner (Fig. 3A). Concomitantly, CASPASE-3/7 activity increased with increasing concentrations of AMPC in both MDA-MB-361 and BT474 cell lines (Fig. 3B). Moreover, the proportion of cells undergoing early and late apoptosis rose with increasing AMPC concentrations in the respective cell lines (Fig. 3C and Supplementary information 4B). In addition, AMPC dose-dependently inhibited MDA-MB-361 and BT474 cell anchorage-independent growth, as demonstrated by foci formation assays (Fig. 3D and Supplementary information 4C). Similarly, the viability of 3D-Matrigel colonies formed by MDA-MB-361 and BT474 cells exhibited a decrement relative to the increasing dose of AMPC. This was evidenced by a discernible decrease in green fluorescence (indicating live cells) and a concomitant increase in red fluorescence (indicating dead cells), as illustrated in Fig. 3E and Supplementary information 4D.

Furthermore, the effect of AMPC on the CSC-like phenotype of MDA-MB-361 and BT474 cells was further examined. Increasing concentrations of AMPC led to a dose-responsive decrease in the size and number of spheroids in both MDA-MB-361 and BT474 cell lines (Fig. 3F and Supplementary information 4E). Consistently, it was observed that AMPC dose-dependently decreased the percentage of the ALDH1-positive cell population in both MDA-MB-361 and BT474 cells (Fig. 3G). These findings indicated that pharmacological inhibition of TFF3 reduced cell viability by suppressing proliferation and inducing apoptosis, and that AMPC is a potent and effective inhibitor of cell proliferation, survival, oncogenicity, 3D-growth, and CSC-like behavior in ER+HER2+MC cells.

### c-MET inhibitors (c-METis) identified as the most synergistic compounds in combination with AMPC to decrease ER+HER2+ MC cell viability

**Fig. 4:**
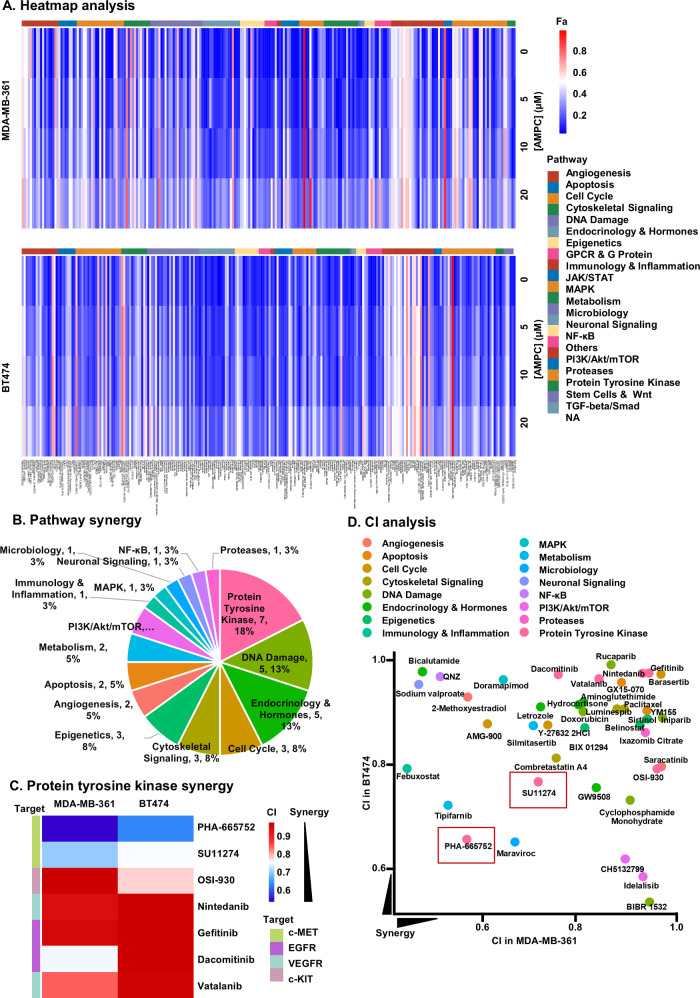
c-METis are identified as the most synergistic compounds in combination with AMPC to decrease ER+HER2+ MC cell viability. **A** Heatmap plot (https://hiplot.com.cn/) depicted the cell viability of MDA-MB-361 and BT474 cells post-treatment as Fraction affected (Fa) (Scale: Blue to Red). The cell viability of cells with the combination treatment of AMPC (0, 5, 10, or 20 µM) with 247 compounds for 3 days was assessed by AlamarBlue (BioChip, Beijing, China). **B** Synergistic pathways with AMPC were statistically analyzed. **C** Heatmap analysis of CI was performed on tyrosine kinase inhibitors (TKIs) exhibiting synergistic effects with AMPC in both MDA-MB-361 and BT474 cells. CI was calculated by Bliss independence method (Bliss CI = (*E*_A_ + *E*_B_-*E*_A_×*E*_B_)/E_AB_). CI < 1 indicated a synergistic effect and a lower CI corresponds to higher synergy. **D** CI analysis was performed on compounds exhibiting synergistic effects with AMPC in both MDA-MB-361 and BT474 cells. CI < 1 indicated a synergistic effect and a lower CI corresponds to higher synergy. The red box highlighted the compounds that exhibited high synergy with AMPC in both MDA-MB-361 and BT474 cells.

Combination therapy improves therapeutic efficacy compared to single-drug treatment by enhancing cytotoxicity and reducing the development of drug resistance in cancer cells [49]. To explore the therapeutic potential of AMPC-based combinations for the treatment of ER+HER2+ MC cells, the Cambridge Anti-Cancer Compound Library was screened in combination with varying concentrations of AMPC (0, 5, 10, or 20 µM) in MDA-MB-361 and BT474 cells, revealing inhibition of cell viability (Fig. 4A). This comprehensive library comprises 247 anti-cancer compounds as illustrated in Fig. 4A. Combination index (CI) analysis [50] revealed that 107 compounds exhibited synergy with AMPC in MDA-MB-361 cells, whereas 79 compounds showed synergy in BT474 cells. Among these, 40 compounds demonstrated synergistic effects with AMPC in both cell lines with detailed information listed in Supplementary information 5. The prominent pathways for the 40 synergistic compounds included protein tyrosine kinase, DNA damage, endocrinology and hormones, cell cycle, cytoskeletal signaling, and epigenetics. Notably, the protein tyrosine kinase pathway was targeted by 7 out of the 40 compounds, comprising 17.5% of all synergistic compounds and ranking first among them (Fig. 4B). Given the role of TFF3 in activating RTKs in ER+HER2+ MC [18], the seven identified compounds in the protein tyrosine kinase pathway primarily targeted four distinct RTKs, c-MET, EGFR, VEGFR, and c-KIT (Fig. 4C). Further scatterplot regression analysis of CI values for compounds demonstrating synergy with AMPC in MDA-MB-361 and BT474 cells revealed that the combination of two c-MET inhibitors, PHA-665752 and SU11274, with AMPC exhibited the highest synergy, characterized by low CI values in both MDA-MB-361 and BT474 cells (Fig. 4D). These findings indicated a synergistic effect of c-MET inhibitors in combination with AMPC in decreasing viability of ER+HER2+ MC cells.

### AMPC synergizes with c-METis to reduce ER+HER2+ MC cell survival and growth

**Fig. 5:**
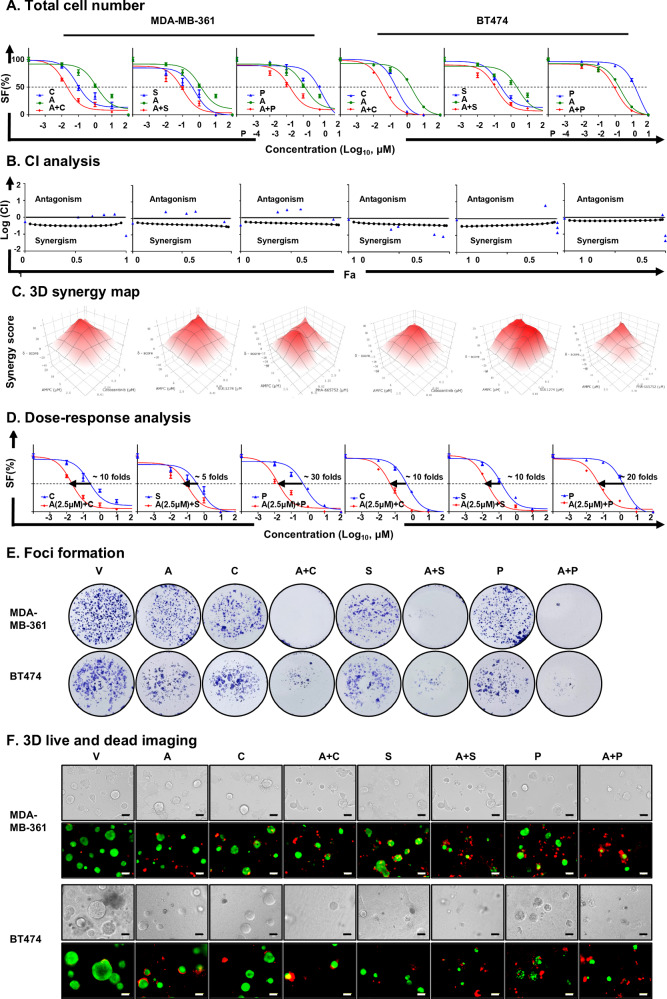
AMPC synergizes with c-METis to reduce ER+HER2+ MC cell survival and growth. **A** MDA-MB-361 and BT474 cells were treated with varying concentration (0.01, 0.1, 1, 10, or 100 μM) of AMPC (A) and Cabozantinib (C), SU11274 (S), or PHA-665752 (P) for 6 days. The survival fraction (SF) in response to AMPC (A) was assessed using a total cell number counting assay. Data are represented as mean ± SD (*n* = 3). **B** CI was determined using the Chou-Talalay method, where CI < 1 indicated synergy, CI = 1 indicated additivity, CI > 1 indicated antagonism. **C** Synergy scores were calculated using bliss synergy analysis (www.synergyfinder.com), where synergy score> 0 indicated synergy, synergy score <0 indicated antagonism. **D** Dose-response analysis of the shift in IC_50_ of Cabozantinib (C), SU11274 (S), or PHA-665752 (P) in MDA-MB-361 and BT474 cells after co-treatment with AMPC (2.5 μM) was conducted with total cell number assay. The arrow indicates the fold reduction in IC_50_ of the respective c-METis in the presence of AMPC. Data are represented as means ± SD (*n* = 3). **E** Foci formation was performed on MDA-MB-361 and BT474 cells treated with vehicle (V), 5 μM AMPC (A), 1 μM Cabozantinib (C), 1 μM SU11274 (S), 2 μM PHA-665752 (P) and the combination in monolayer culture at low cell density for 14 days. Foci were stained by crystal violet. **F** MDA-MB-361 and BT474 cells were treated with vehicle (V), 5 μM AMPC (A), 1 μM Cabozantinib (C), 1 μM SU11274 (S), 2 μM PHA-665752 (P), and the combination in medium containing 2% FBS and 4% Matrigel for 12 days after 3 days of pre-culture. Bright-field images showed the colonies, while merged images depicted Live/Dead staining, with red indicating dead colonies and green indicating live colonies. Scale bars, 100 μm.

To further substantiate the synergistic effects of AMPC and c-METis identified through high-throughput screening of the anti-cancer compound library, the pharmacological inhibition of TFF3 by AMPC in combination with three c-METis was further evaluated in MDA-MB-361 and BT474 cells by total cell number assay (Fig. 5A). Cabozantinib, an FDA-approved c-METi not included in the Cambridge Anti-Cancer Compound Library (Supplementary information 6A), alongside the identified PHA-665752 and SU11274, were chosen for continued investigation. The combinatorial treatments of AMPC and c-METis exhibited synergistic effects, as demonstrated by the Chou-Talalay method and 3D zip synergy analysis in both cell lines (Fig. 5B, C). Subsequently, combination treatment of AMPC (2.5 μM)—c-METis significantly increased the efficacy of c-METis compared to c-METi treatment alone in MDA-MB-361 and BT474 cells, as demonstrated by dose-response analysis (Fig. 5D). Specifically, AMPC reduced IC_50_ values of Cabozantinib, SU11274, and PHA-665752 by approximately 10-fold, 5-fold, and 30-fold, respectively, in MDA-MB-361 cells (Fig. 5D). Similarly, in BT474 cells, AMPC notably decreased the IC_50_ values of Cabozantinib, SU11274 and PHA-665752 by approximately 10-fold, 10-fold, and 20-fold, respectively (Fig. 5D). These findings underscore the synergistic potential of combining AMPC with c-METis to decrease cell survival in ER+HER2+ MC cells.

Furthermore, the effect of the combinatorial treatment on cell proliferation and apoptosis was investigated. The results indicated that single-agent AMPC or c-METis significantly inhibited proliferative capability compared to vehicle treatment in both MDA-MB-361 and BT474 cells (Supplementary information 7A). Importantly, the combined AMPC-c-METi treatments further amplified the inhibitory effect observed with the single treatments. Additionally, single-agent AMPC or c-METis promoted CASPASE 3/7 activity compared to vehicle treatment in both cell lines, and combined AMPC-c-METi treatments further enhanced this effect (Supplementary information 7B). This observation was consistent with an increase in apoptosis, as evidenced by elevated populations of early and late apoptotic cells upon single-agent and combined AMPC-c-METi treatments in both cell lines (Supplementary information 7C).

Subsequently, the effect of combined AMPC-c-METi treatments on foci-forming capacity and 3D growth of MDA-MB-361 and BT474 cells was evaluated. Foci formation assays demonstrated that single treatments with AMPC or c-METis significantly reduced colony formation and viability compared to the vehicle control. Notably, the combined treatment of AMPC and c-METis further augmented these effects observed with the individual treatments (Fig. 5E and Supplementary information 7D). Similarly, in 3D Matrigel culture, single treatments led to a substantial reduction in the number and size of colonies, decreased cell viability, increased numbers of dead cells, and fewer live cells compared to the vehicle control. These effects were more pronounced with the combined treatments (Fig. 5F and Supplementary information 7E). Collectively, these findings indicate that AMPC synergizes with c-MET inhibitors to significantly reduce the survival and growth of ER+HER2+ MC cells in vitro and in 3D matrigel.

### AMPC synergizes with c-METis to suppress CSC-like phenotype in ER+HER2+ MC cells

**Fig. 6:**
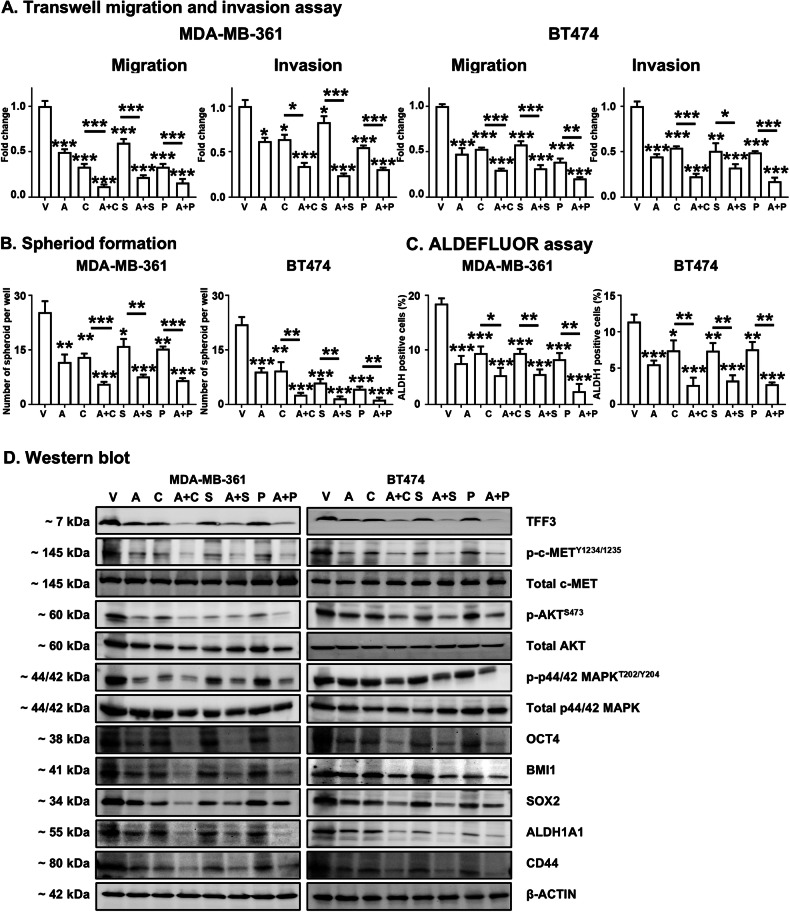
AMPC synergizes with c-METis to suppress CSC-like phenotype in ER+HER2+ MC cells*.* **A** Cell migration and invasion assays were conducted on MDA-MB-361 and BT474 cells treated with vehicle (V), 5 μM AMPC (A), 1 μM Cabozantinib (C), 1 μM SU11274 (S), 2 μM PHA-665752 (P) or the combination. Data are expressed as mean ± SD (*n* = 3). Statistical significance is indicated as *P < 0.05, **P < 0.01, and ***P < 0.001. **B** MDA-MB-361 and BT474 cells were seeded in ultralow attachment plates and cultured in spheroid growth media, and treated with vehicle (V), 5 μM AMPC (A), 1 μM Cabozantinib (C), 1 μM SU11274 (S), 2 μM PHA-665752 (P), or the combination for 12 days. The spheroids with diameters greater than 50 μm in each well were counted. Data are expressed as mean ± SD (*n* = 3). Statistical significance is indicated as *P < 0.05, **P < 0.01, and ***P < 0.001. **C** ALDEFLUOR activity was measured after 3 days of treatment with vehicle (V), 5 μM AMPC (A), 1 μM Cabozantinib (C), 1 μM SU11274 (S), 2 μM PHA-665752 (P), or the combination. MDA-MB-361 and BT474 cells were then harvested and incubated with ALDEFLUOR substrate to define the ALDH1-positive population, with DEAB used to establish the baseline fluorescence. Data are expressed as mean ± SD (*n* = 3). Statistical significance is indicated as *P < 0.05, **P < 0.01, and ***P < 0.001. **D** Western blot analysis was conducted to assess the expression and/or phosphorylation of TFF3, c-MET, AKT, p44/42 MAPK and CSC-related proteins in MDA-MB-361 and BT474 cells following treatment with vehicle (V), 5 μM AMPC (A), 1 μM Cabozantinib (C), 1 μM SU11274 (S), 2 μM PHA-665752 (P), or the combination for 3 days. β-ACTIN was used as input control. The sizes of detected proteins in kDa are indicated on the left.

To assess the effect of the combinatorial treatment on the migration and invasion of ER+HER2+ MC cells, the migratory and invasive capacities were evaluated following single treatments with AMPC, c-METis, and their combination in MDA-MB-361 and BT474 cells. The findings revealed that single-agent treatments notably suppressed both the migratory and invasive abilities of the cells compared to the control treatment (Fig. 6A and Supplementary information 8A). The combined AMPC-c-METi treatments exhibited an augmented inhibition effect beyond that of the single-agent treatments, suggesting a synergistic enhancement in restraining the migratory and invasive capacities of the ER+HER2+ MC cells.

There exists a positive relationship between the size of the subpopulation of MC CSCs and migration and invasion [51]. Hence, the spheroid formation and ALDEFFLOUR activity assays were conducted to explore the effect of AMPC and c-METis combined treatment on CSC-like behavior in ER+HER2+ MC cells. The results from spheroid formation assay indicated that single treatments with AMPC or c-METis significantly reduced the number of spheroids compared to vehicle treatment, and the combined AMPC-c-METi treatments further diminished spheroid formation in both ER+HER2+ MC cell lines (Fig. 6B and Supplementary information 8B). Consistently, the percentage of the ALDH1-positive cell population decreased after treatment with either AMPC or c-METis alone compared to the vehicle control (Fig. 6C and Supplementary information 8C). Notably, the combined AMPC-c-METi treatments resulted in a significant reduction in the ALDH1-positive cell population compared to c-METi treatment alone (Fig. 6C and Supplementary information 8C).

Subsequently, the mechanistic basis underlying synergistic effects of AMPC and c-METis was further analyzed using western blot analysis. Single treatments with either AMPC or c-METis, decreased TFF3 expression, and the p-c-MET^Y1234/1235^/c-MET ratio in MDA-MB-361 and BT474 cells; and combined AMPC and c-METi treatments further significantly reduced TFF3 expression and the p-c-MET^Y1234/1235^/c-MET ratio compared to single agent treatment of ER+HER2 + MC cells (Fig. 6D and Supplementary information 8D). As downstream pathways of TFF3 and c-MET, the phosphorylation (activity) of AKT and p44/42 MAPK decreased after single treatments with either AMPC or c-METis, and combined AMPC and c-METi treatments further reduced p-AKT^S473^/AKT and p-p44/42 MAPK^T202/Y204^/p44/42 MAPK ratios (Fig. 6D and Supplementary information 8D). Previous studies have shown that CSCs express elevated levels of genes related to migration and invasion, such as OCT4, BMI1, SOX2, ALDH1A1, and CD44 in MC [52–55]. Diminished expression of OCT4, BMI1, SOX2, ALDH1A1, and CD44 proteins was observed in MDA-MB-361 and BT474 cells treated with AMPC or c-METis, in comparison to vehicle-treated cells (Fig. 6D and Supplementary information 8D). These reductions were further magnified following combined AMPC-c-METi treatments (Fig. 6D and Supplementary information 8D). Thus, the combined treatment of AMPC with c-METis demonstrated significant potential to synergistically inhibit the CSC-like phenotype of ER+HER2+ MC cells.

### AMPC synergizes with c-MET inhibition to suppress MDA-MB-361 xenograft growth and lung metastasis

Building on the synergistic effects observed in vitro, the efficacy of the combined treatment of AMPC and c-MET inhibition was further evaluated in vivo. The xenograft model was established by orthotopically implanting MDA-MB-361 cells into the right fourth mammary fat pad of female mice. Cabozantinib was selected for the in vivo experiments as it is the FDA-approved drug for c-MET inhibition, and has been utilized in patients with ER+ MC or HER2+ MC for two Phase II clinical trials [56, 57]. Mice bearing MDA-MB-361 xenografts were randomized into four treatment groups (*n* = 8) to receive either vehicle (V), AMPC (A), Cabozantinib (C), or a combination of AMPC and Cabozantinib (A + C). Each treatment was administered daily for 2 weeks. Throughout the treatment period, animal weights and xenograft volumes were measured daily. Upon completion of the treatment regimen, major organs, including the spleen, lungs, and liver, were collected post-mortem. There was no significant difference in body weight or major organ weights among the four treatment groups, indicating that the treatments were well-tolerated by the mice (Supplementary information 9A, 9B).

**Fig. 7:**
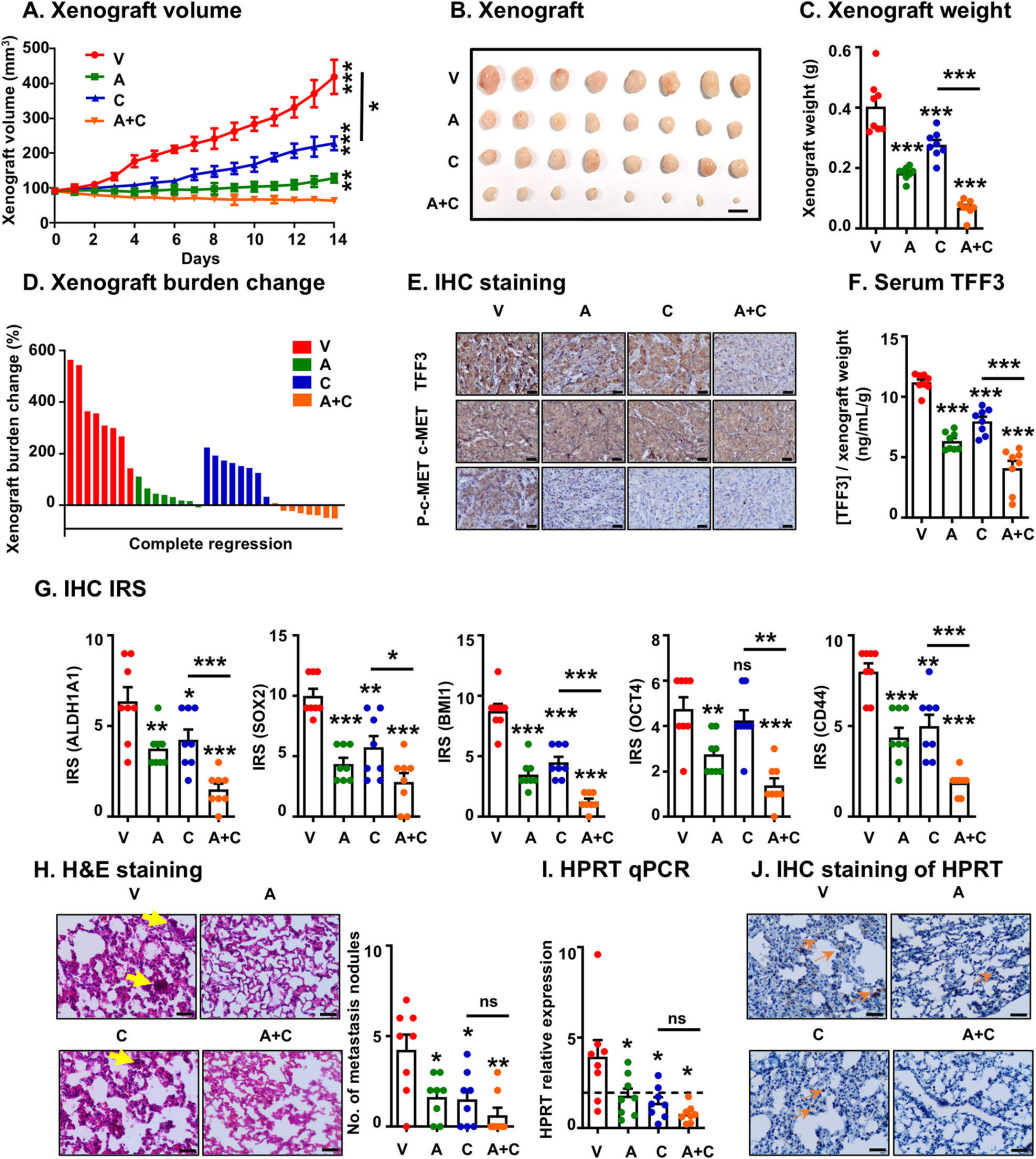
AMPC synergizes with a c-METi to suppress MDA-MB-361 xenograft growth and lung metastasis. **A** The volumes of MDA-MB-361 xenografts (mm^3^) were measured daily and calculated using the formula: 0.52 × length × [width]^2^ for the treatments with vehicle (V), 20 mg/kg AMPC (A), 60 mg/kg Cabozantinib (C) and the combination of AMPC and Cabozantinib (A + C). Data are expressed as mean ± SD (*n* = 8). Statistical significance is indicated as *P < 0.05, **P < 0.01, and ***P < 0.001. **B** Resected MDA-MB-361 xenografts of each treatment group at the termination of the experiments (*n* = 8), Scale bar, 1 cm. **C** Weights of resected MDA-MB-361-derived xenografts were determined at the termination of the experiments. Data are expressed as mean ± SD (*n* = 8). Statistical significance is indicated as *P < 0.05, **P < 0.01, and ***P < 0.001. **D** Xenograft burden change of MDA-MB-361 xenografts for each treatment group was calculated after sacrificing at the termination of the experiments. **E** Representative micrographs of IHC staining for TFF3 and c-MET in the indicated xenografts. Scale bar, 20 μm. **F** ELISA analysis was conducted to measure serum levels of human TFF3 derived from xenografts. Data are expressed as mean ± SD (*n* = 8). Statistical significance is indicated as *P < 0.05, **P < 0.01, and ***P < 0.001. **G** IRS score of ALDH1A1, SOX2, BMI1, and OCT4 was assessed. Data are expressed as mean ± SD (*n* = 8). Statistical significance is indicated as *P < 0.05, **P < 0.01, and ***P < 0.001. **H** H&E staining and quantitative measurement of micrometastatic nodules were performed in lungs of MDA-MB-361 xenograft model. Scale bar, 20 μm. Data are expressed as mean ± SD (*n* = 8). Statistical significance is indicated as *P < 0.05, **P < 0.01, and ***P < 0.001. **I** mRNA expression of human HPRT gene in the lungs at the termination of the experiments was detected. The dotted line represented the baseline expression of the human HPRT gene in mice without intravenous injection of MDA-MB-361 cells. Data are expressed as mean ± SD (*n* = 8). Statistical significance is indicated as *P < 0.05, **P < 0.01, and ***P < 0.001. **J** Representative micrographs of IHC staining for HPRT were shown in the indicated lungs. Scale bar, 20 μm.

Daily assessments of xenograft volumes revealed that single-agent treatments with either AMPC or Cabozantinib significantly reduced the volumes of MDA-MB-361 xenografts compared to the vehicle-treated control. Notably, the combination treatment with AMPC and Cabozantinib resulted in an even greater reduction in xenograft volumes compared to either single-agent treatment alone (Fig. 7A). Consistent results were observed with xenograft weight (Fig. 7B) and resected xenografts (Fig. 7C). Moreover, as demonstrated by xenograft burden change and mRECIST analysis (Fig. 7D and Supplementary information 9C), whereas single-agent treatments with AMPC or Cabozantinib failed to achieve complete or partial responses in MDA-MB-361 xenografts, a proportion of xenografts treated with AMPC (37.5%) or Cabozantinib (12.5%) exhibited stable disease (mSD), contrasting with xenografts treated with the vehicle (0.0%), which showed progression (mPD). Remarkably, xenografts treated with the combination of AMPC and Cabozantinib displayed a partial response (mPR) in 12.5% of cases and mSD in 87.5% of cases. Furthermore, IHC and TUNEL analyses revealed a decreased proportion of MKI67-positive cells, and a higher incidence of apoptosis in the MDA-MB-361 xenograft specimens treated with single-agent treatments compared to those treated with the vehicle control (Supplementary information 9D, 9E). These effects were further enhanced in specimens treated with the combination of AMPC and Cabozantinib. Therefore, these results collectively provide evidence of the increased effectiveness of the combination treatment in suppressing MDA-MB-361 xenograft growth and halting disease progression.

To further investigate the effects of the combined treatment on CSCs in vivo, IHC coupled with IRS score analysis was conducted on xenograft specimens. The results showed a decrease in TFF3 protein levels and concurrently decreased phosphorylation of c-MET in xenograft tissues after treatment with single-agent AMPC or Cabozantinib. The decreased phosphorylation of c-MET was significantly amplified when the AMPC-Cabozantinib combination was administered (Fig. 7E and Supplementary information 9F). Moreover, the diminished expression of TFF3 was validated by analyzing the relative levels of serum TFF3 in comparison to xenograft weight (Fig. 7F). Subsequently, the effect of combined targeting on CSC markers in the xenograft specimens was conducted. A significant decrease in the expression of CSC markers OCT4, BMI1, SOX2, ALDH1A1, and CD44 in xenograft samples from the groups treated with single drugs compared to the vehicle-treated control group was observed (Fig. 7G and Supplementary information 9G). The group receiving the combination treatment demonstrated a further significant suppression of the expression of these markers in comparison to the individual drug treatments.

Next, the potential metastatic dissemination of MDA-MB-361 cells from the xenograft site to major organs was determined. Metastasis was first assessed in lung tissue sections using hematoxylin-eosin (H&E) staining (Fig. 7H and Supplementary information 9H). The H&E results revealed that there was reduced incidence of lung metastasis in both single-agent AMPC (6/8) and Cabozantinib (5/8) treated groups compared to the vehicle-treated group (7/8). Notably, the incidence of lung metastasis was dramatically reduced in the combined AMPC-Cabozantinib treated group (2/8). Quantification of metastatic nodules in the lungs further corroborated this reduction (Fig. 7H). Moreover, the human HPRT gene (*hHPRT*) was utilized to distinguish the metastatic burden of cells of human origin, as previously reported [44, 58]. The relative expression of *hHPRT* to *mgapdh* verified a significantly decreased metastatic burden in both single-agent AMPC and Cabozantinib-treated groups compared to the vehicle-treated group (Fig. 7I). The reduced metastatic burdens were further confirmed through IHC and IRS score analysis of hHPRT protein in lungs from the different treatment groups (Fig. 7J). Collectively, these findings demonstrate the enhanced efficacy of the combined treatment in mitigating micrometastatic spread.

In summation, combined treatment employing AMPC and Cabozantinib as a therapeutic strategy effectively controlled the growth of primary xenografts and reduced the tendency for lung metastasis in a ER+HER2+ MC xenograft model.

### TFF3 enhances c-MET signaling through a positive feedback loop to enhance the CSC-like phenotype of ER+HER2+ MC

**Fig. 8:**
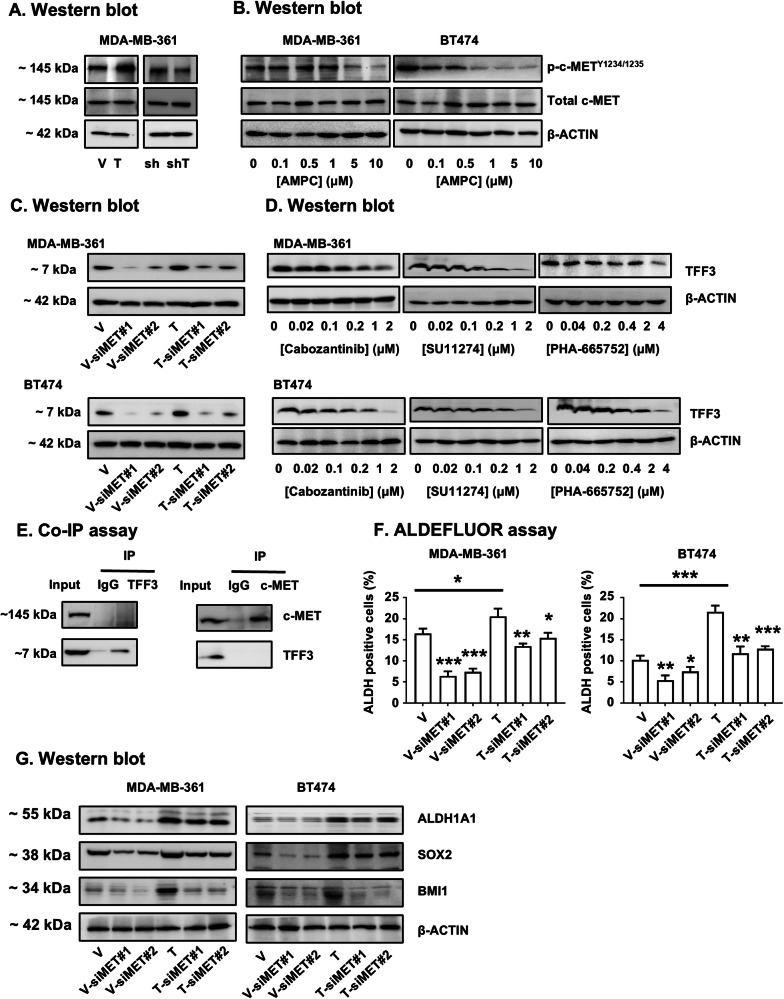
TFF3 enhances c-MET signaling through a positive feedback loop to enhance the CSC-like phenotype of ER+HER2+ MC. **A** Western blot analysis was conducted to assess the level of c-MET and c-MET phosphorylation at Y1234/1235 in MDA-MB-361 cells with TFF3 forced expression (V & T) and depletion (sh & shT). β-ACTIN was used as input control. The sizes of detected protein blots in kDa are on the left. **B** Western blot analysis was conducted to assess the level of c-MET and c-MET phosphorylation at Y1234/1235 in MDA-MB-361 and BT474 cells with AMPC (0, 0.1, 0.5, 1, 5, or 10 μM) treatment for 3 days. β-ACTIN was used as input control. The sizes of detected protein blots in kDa are shown on the left. **C** Western blot analysis was performed to assess the level of TFF3 in MDA-MB-361 and BT474 cells transfected with scrambled siRNA, siMET#1, or siMET#2 plasmid. β-ACTIN was used as input control. The sizes of detected protein blots in kDa are shown on the left. **D** Western blot analysis was performed to assess the level of TFF3 in MDA-MB-361 and BT474 cells treated with Cabozantinib (0, 0.02, 0.1, 0.2, 1 or 2 μM), SU11274 (0, 0.02, 0.1, 0.2, 1 or 2 μM) or PHA-665752 (0, 0.04, 0.2, 0.4, 2 or 4 μM) for 3 days. β-ACTIN was used as input control. The sizes of detected protein blots in kDa are shown on the left. **E** A potential c-MET/TFF3 interaction in MDA-MB-361 cells was investigated by immunoprecipitation (IP) and immunoblotting. **F** MDA-MB-361 and BT474 cells transfected with scrambled siRNA, siMET#1, or siMET#2 plasmid were harvested and incubated with ALDEFLUOR substrate to define the ALDH1-positive population. DEAB was used to establish the baseline fluorescence. Data are expressed as mean ± SD (*n* = 3). Statistical significance is indicated as *P < 0.05, **P < 0.01, and ***P < 0.001. **G** Western blot analysis was utilized to examine the level of CSC-related proteins in MDA-MB-361 and BT474 cells transfected with scrambled siRNA, siMET#1, or siMET#2 plasmid. β-ACTIN was used as input control. The sizes of detected protein blots in kDa are shown on the left.

A previous study has reported that the forced expression of TFF3 enhanced various RTK activities in ER+HER2+ MC, including c-MET, suggesting a potential regulatory relationship between TFF3 and c-MET [18]. To delineate this potential relationship, the phosphorylation of c-MET was assessed in MDA-MB-361 and BT474 cells. Western blot analysis revealed that the phosphorylation levels of c-MET at Tyrosine 1234/1235 were significantly elevated in MDA-MB-361 cells with forced expression of TFF3 compared to the vector-transfected control (Fig. 8A and Supplementary information 10D). Conversely, the c-MET phosphorylation levels were markedly decreased in MDA-MB-361 cells with TFF3 depletion compared to control vector cells (Fig. 8A and Supplementary information 10D). Moreover, pharmacological inhibition of TFF3 by AMPC induced a dose-dependent decrease in c-MET phosphorylation at Tyrosine 1234/1235 in MDA-MB-361 and BT474 cells (Fig. 8B and Supplementary information 10E).

Given the intricate interplay previously reported between HER2 and c-MET [59, 60], alongside their shared attributes as tyrosine receptor kinases and the findings from combination experiments herein demonstrating the suppressive effect of c-METis on TFF3 expression both in vitro and in vivo (Figs. 6D, 7E); whether there is a regulatory association between TFF3 and c-MET was therefore further investigated. It was observed that the expression of TFF3 was markedly suppressed in MDA-MB-361 and BT474 cells following c-MET depletion using two independent siRNAs targeting c-MET (siMET#1 and siMET2#2), when compared to the respective scrambled siRNA transfected cells (Fig. 8C and Supplementary information 10A, F and I). Furthermore, the expression of TFF3 exhibited a dose-dependent reduction upon treatment with c-METis in both cell lines (Fig. 8D and Supplementary information 10G). Since c-MET also appears to be functionally downstream of TFF3 activated pathways, co-IP assays were conducted to investigate whether TFF3 itself may be an alternate ligand for c-MET. However, no association of TFF3 to c-MET was observed in MDA-MB-361 cells, whereas the reported c-MET ligand HGF demonstrated the interaction between c-MET and HGF as a positive control (Fig. 8E and Supplementary information 10B). Therefore, the phosphorylation of c-MET enhanced by TFF3 may occur indirectly, as for other RTKs [26, 61, 62].

The synergistic inhibition of TFF3 by AMPC combined with c-METis effectively decreased the CSC-like phenotype in ER+HER2+ MC cells both in vitro and in vivo (Fig. 6C and Supplementary information 7G). To further explore the effect of the TFF3-c-MET pathway on CSC-like behavior, ER+HER2+ MC cells with forced expression of TFF3 and with c-MET depletion were examined by western blot and ALDEFLUOR assays. The elevated population of ALDH1-positive cells observed in cells with forced expression of TFF3 was significantly reduced following c-MET depletion compared to the scrambled siRNA transfected cells (Fig. 8F and Supplementary information 10C). Western blot analyses demonstrated that the increased expression of CSC markers BMI1, SOX2, and ALDH1A1 in both MDA-MB-361 and BT474 cells with forced expression of TFF3 was mitigated upon c-MET depletion (Fig. 8G and Supplementary information 10H). Collectively, it is apparent that c-MET positively regulates its own signaling through TFF3 with consequent enhancement of the CSC-like phenotype in ER+HER2+ MC cells.

## Discussion

Despite significant advances in treatments for both HER2+ and ER+ MC, and the resultant survival benefits for affected patients, ER+HER2+ MC remains an underrepresented subgroup lacking sufficient tailored therapeutic options due to its distinct characteristics from either HER2+ or ER+ MC [63, 64]. Over the past decade, TFF3 has emerged as a promising therapeutic target due to its promotory role in cancer progression, including colorectal, hepatocellular, lung, pancreatic, prostate, cervical, endometrial, and ER+ mammary carcinomas [9, 10, 12–21]. Furthermore, inhibition of TFF3 has been shown to enhance the efficacy of ionizing radiation, Gemicitabine, Taxanes, and MEK1/2 inhibitors, and to overcome resistance to 5-FU, anti-estrogen, and HER2-targeted therapy [9, 12, 18]. Previous studies have reported that the pharmacological inhibition of TFF3 modulates the PI3K/AKT, MAPK, WNT, and JAK/STAT3 signaling pathways in carcinoma cells [9, 12, 13, 20, 30]. Consistently, this study revealed that TFF3 possesses an oncogenic role in ER+HER2+ MC and demonstrated that TFF3 inhibition enhances the effectiveness of inhibition of c-MET. These findings suggest TFF3 as a novel promising target for combination therapeutic strategies in ER+HER2+ MC.

CSCs are a critical subpopulation of tumor-initiating cells implicated in cancer relapse, metastasis, and resistance to radiotherapy and chemotherapy [65]. BCSCs were initially recognized based on the relative expression of CD44 and CD24 [66]. CD44, CD24, and aldehyde dehydrogenase-1 (ALDH1) are now widely used as biomarkers for identifying BCSC characteristics [67]. One of the defining characteristics of ER+HER2+ MC is the presence of ALDH1+ epithelial BCSCs, which is associated with poor clinical prognosis [3]. Moreover, BCSCs have been implicated in the failure of endocrine therapy, chemotherapy, radiotherapy, and immunotherapy in MC treatment, ultimately promoting relapse [68]. Recently, several studies have reported that TFF3 promotes a CSC-like phenotype in pancreatic, colorectal, hepatocellular, lung, cervical, and ER+ mammary carcinoma [10, 12, 13, 15, 18–20]. In ER+ MC patients, expression and activation of c-MET is significantly higher in metastatic sites than in primary sites [69]. Furthermore, c-MET expression has been strongly correlated with CSC markers, ALDH1A3 and CD133 in MC [70]. High c-MET expression and its activation are also suggested to be involved in the promotion of *ALDH1A3* gene expression in the basal-like type of MC [70]. This investigation delineated the role of TFF3 in enhancing CSC-like phenotype in ER+HER2+ MC cells, as evidenced by heightened ALDH1 activity and increased spheroid formation capacity (Fig. 1G, H). Additionally, this study revealed that inhibiting TFF3 reduced CSC-like phenotype, and dual inhibition of TFF3 and c-MET led to a further reduction, suggesting a potential mechanism for the decreased metastatic burden observed with combinatorial therapy of AMPC and c-METis in ER+HER2+ MC.

Intriguingly, the high-throughput anti-cancer compound screening assays demonstrated that TFF3 inhibition by AMPC synergized most effectively with compounds targeting four distinct RTKs, EGFR, VEGFR, c-KIT, and c-MET in MDA-MB-361 and BT474 cells. These RTKs have all been implicated in oncogenic progression and are potential targets for cancer therapy. Inhibiting EGFR, VEGFR, and c-MET, whether by single drugs or drug combinations, has proven beneficial by halting cancer cell growth, proliferation, and metastasis [71, 72] although resistance ultimately develops. Mechanistically, it has been previously shown that TFF3 can competitively bind with LINGO2 to disrupt EGFR-LINGO2 complexes, leading to the release of EGFR activity [73]. Additionally, targeting TFF3 with AMPC resulted in decreased EGFR activity in ER+ MC cells [30]. The capacity of TFF3 to enhance the activation of the EGFR further indicates the importance of TFF3 as a therapeutic target in that TFF3 may modulate other RTKs through its involvement in multiple signaling pathways, including p44/42 MAPK [9], PI3K/AKT [28, 29] and STAT3 [15, 16]. Given the reported functions of TFF3 in modulating RTK-mediated cellular functions [18, 30], including the data herein, it may be thus reasoned that RTK inhibition in cancer will be rendered more efficacious by TFF3 depletion or inhibition.

To date, no FDA-approved c-MET inhibitor exists for MC, however, clinical investigations are currently underway to assess the effectiveness of c-MET-targeted therapies in MC patients. Cabozantinib is a multi-kinase inhibitor targeting c-MET, VEGFR1-3, RET, AXL, FLT3, and c-KIT [71]. In a single-arm Phase II study recruited patients with ER+ MC and bone metastases treated with daily Cabozantinib (NCT01441947) demonstrated efficacy of Cabozantinib [57]. The clinical benefits of Cabozantinib were also explored in ER+ MC and HER2+ MC patients with brain metastases (NCT02260531) in a Phase II trial [74]. Clinical trials involving c-MET-targeted medications in MC have exhibited varied outcomes suggesting that a combination strategy with c-MET inhibition in ER+HER2+ MC may be more useful.

In ER+HER2+ MC, increased expression of TFF3 has been implicated in trastuzumab resistance, activating both the HER family of tyrosine kinases and crosstalk partners, including c-MET [18]. Moreover, crosstalk of c-MET signaling pathways with ER and HER2 signaling pathways has been reported [75, 76]. Hiscox *et al*. reported the increased expression of c-MET along with a marked increase in the migratory and invasive capacity of Fulvestrant-resistant MC cells, and observed that increased expression of c-MET in endocrine therapy-resistant epithelial MC cells promoted cancer progression [75]. Furthermore, Shattuck et al. reported that c-MET is frequently co-expressed with HER2 in HER2+ MC and contributes to trastuzumab resistance of HER2+ MC cells through sustained AKT activation; whereas the loss of c-MET function, either through RNA interference-mediated depletion or small molecule-mediated inhibition, significantly improves the response to trastuzumab [76]. c-MET enhances the activation of PI3K/AKT and p44/42 MAPK signaling, two downstream signaling pathways also enhanced by TFF3 [7], and which display heightened activity in ER+HER2+ MC and in lymph node metastases of this MC subtype [7, 71, 77]. This may explain the lack of a complete response observed for xenograft growth and lung metastasis in xenograft models with the combination treatment of AMPC and Cabozantinib (Fig. 7A and Supplementary information 9H). However, given the observed efficacy, it may be postulated that prolonging the treatment duration or optimizing the dosage/dose intensity may markedly enhance the therapeutic response. Additionally, supplementing with a third drug, such as selective estrogen receptor modulators or HER2 targeting agents, might provide a more efficacious approach to treating ER+HER2+ MC and improving patient outcomes.

Consistent with a previous study for HER2 [18], it is herein hypothesized that c-MET regulates its signaling through a positive feedback loop by TFF3 in ER+HER2+ MC cell lines. However, co-immunoprecipitation assays failed to demonstrate direct binding of TFF3 and c-MET (Fig. 8E). This is consistent with other RTKs, as TFF3-stimulated EGFR activation was achieved without direct binding or colocalization of TFF3 and EGFR [61, 62]. A possible mechanism through which TFF3 activates c-MET in ER+HER2+ MC is activation via crosstalk pathways including HER2, as TFF3 has been previously shown to activate HER2, a demonstrated heterodimeric partner of c-MET [18, 78, 79]. There may also exist a mechanism analogous to TFF3-LINGO2-EGFR [26] in which TFF3 sequesters proteins interacting with c-MET that inhibit its activation.

The investigations herein highlight the pivotal role of TFF3 in the oncogenicity of ER+HER2+ MC cells and disease progression. This study therefore enhances the understanding of HER2+ER+ MC progression by delineating the bidirectional control mechanisms involving c-MET and TFF3 in ER+HER2+ MC cells. TFF3 enhanced phosphorylation of c-MET and c-MET signaling was abrogated by TFF3 inhibition or depletion. Furthermore, TFF3 expression was increased by c-MET activation, and decreased by inhibition of c-MET. Hence, the complex molecular landscape of this subtype has been further clarified by the delineation of the interactions of TFF3 and c-MET signaling pathways, offering insight into the potential amelioration of targeted therapy for ER+HER2+ MC. The collective evidence suggests that targeting TFF3 and c-MET represents a promising and potentially efficacious treatment approach for addressing the unique challenges posed by ER+ HER2+ MC.

## Supplementary information

Supplementary information

Supplementary information for uncropped western blot images

## Data Availability

The data sets used in this study are available from the corresponding author upon reasonable request.
