# Supplementary material for: Inhibition of TFF3 synergizes with c-MET inhibitors to decrease the CSC-like phenotype and metastatic burden in ER+HER2+ mammary carcinoma
Source: Cell Death Dis. 2025 Feb 7;16(1):76. doi: 10.1038/s41419-025-07387-5 (PMC11806102; doi:10.1038/s41419-025-07387-5)
Supplement: Supplementary file 1 — Supplementary information [file 41419_2025_7387_MOESM1_ESM.docx]

**Supplementary information for**

**Inhibition of TFF3 Synergizes with c-MET Inhibitors to Decrease the CSC-like Phenotype and Metastatic Burden in ER+HER2+ Mammary Carcinoma**

Chuyu He^1,7^, Xuejuan Wang^1,7^, Yi-Shiou Chiou^1,2^, Basappa Basappa^3^, Tao Zhu^4,5,6^, Vijay Pandey^1*^ and Peter E. Lobie^1, 4*^

^1^ Institute of Biopharmaceutical and Health Engineering and Tsinghua Berkeley Shenzhen Institute, Tsinghua Shenzhen International Graduate School, Tsinghua University, Shenzhen 518055, People’s Republic of China.

^2^Master Degree Program in Toxicology, College of Pharmacy, Kaohsiung Medical University, Kaohsiung, 807, Taiwan.

^3^Laboratory of Chemical Biology, Department of Studies in Organic Chemistry, University of Mysore, Manasagangotri 570006 Mysore, India.

^4^Shenzhen Bay Laboratory, Shenzhen 518055 Guangdong, People’s Republic of China.

^5^Department of Oncology, The First Affiliated Hospital of USTC, Center for Advanced Interdisciplinary Science and Biomedicine of IHM, Division of Life Sciences and Medicine, University of Science and Technology of China, Hefei, Anhui 230027, People’s Republic of China.

^6^Hefei National Laboratory for Physical Sciences, University of Science and Technology of China, Hefei, Anhui 230027, People’s Republic of China.

^7^These authors contributed equally to this work.

* Correspondence: [vijay.pandey@sz.tsinghua.edu.cn](mailto:vijay.pandey@sz.tsinghua.edu.cn); [pelobie@sz.tsinghua.edu.cn](mailto:pelobie@sz.tsinghua.edu.cn)

**Supplementary information 1**


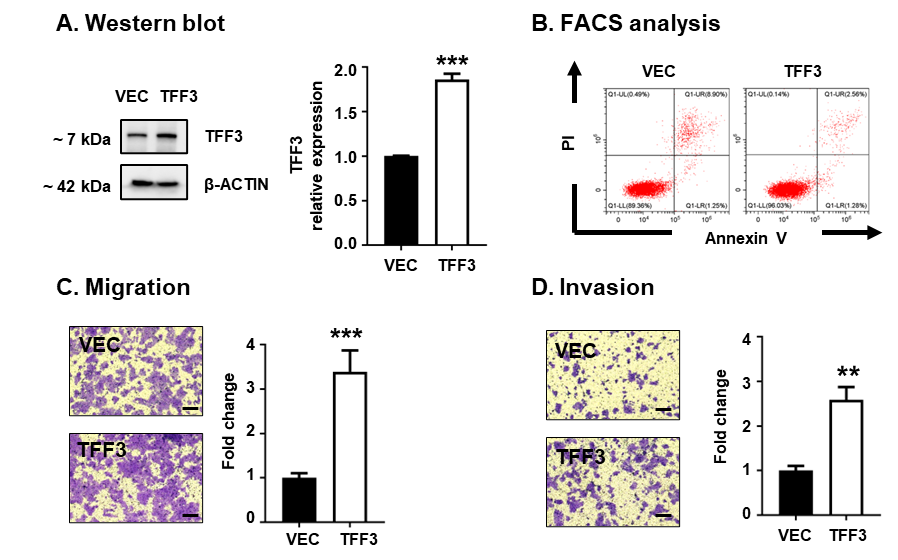


**A.** Western blot analysis was performed to determine the expression of TFF3 in MDA-MB-361-VEC (VEC) and MDA-MB-361-TFF3 (TFF3) cells. Densitometric analysis of western blots was conducted by using ImageJ software. Data are expressed as mean ± SD (*n* = 3). Statistical significance is indicated as **P* < 0.05, ***P* < 0.01, and ****P* < 0.001.

**B.** The percentage of early apoptotic (Annexin-V-positive/PI negative) and late apoptotic (Annexin-V-positive/PI-positive) cells was plotted by flow cytometry in MDA-MB-361-TFF3 (TFF3) and MDA-MB-361-VEC (VEC) cells.

**C.** Cell migration assay. Scale bar, 50 μm. Data are expressed as mean ± SD (*n* = 3). Statistical significance is indicated as **P* < 0.05, ***P* < 0.01, and ****P* < 0.001.

**D.** Cell invasion assay. Scale bar, 50 μm. Data are expressed as mean ± SD (*n* = 3). Statistical significance is indicated as **P* < 0.05, ***P* < 0.01, and ****P* < 0.001.

**Supplementary information 2**


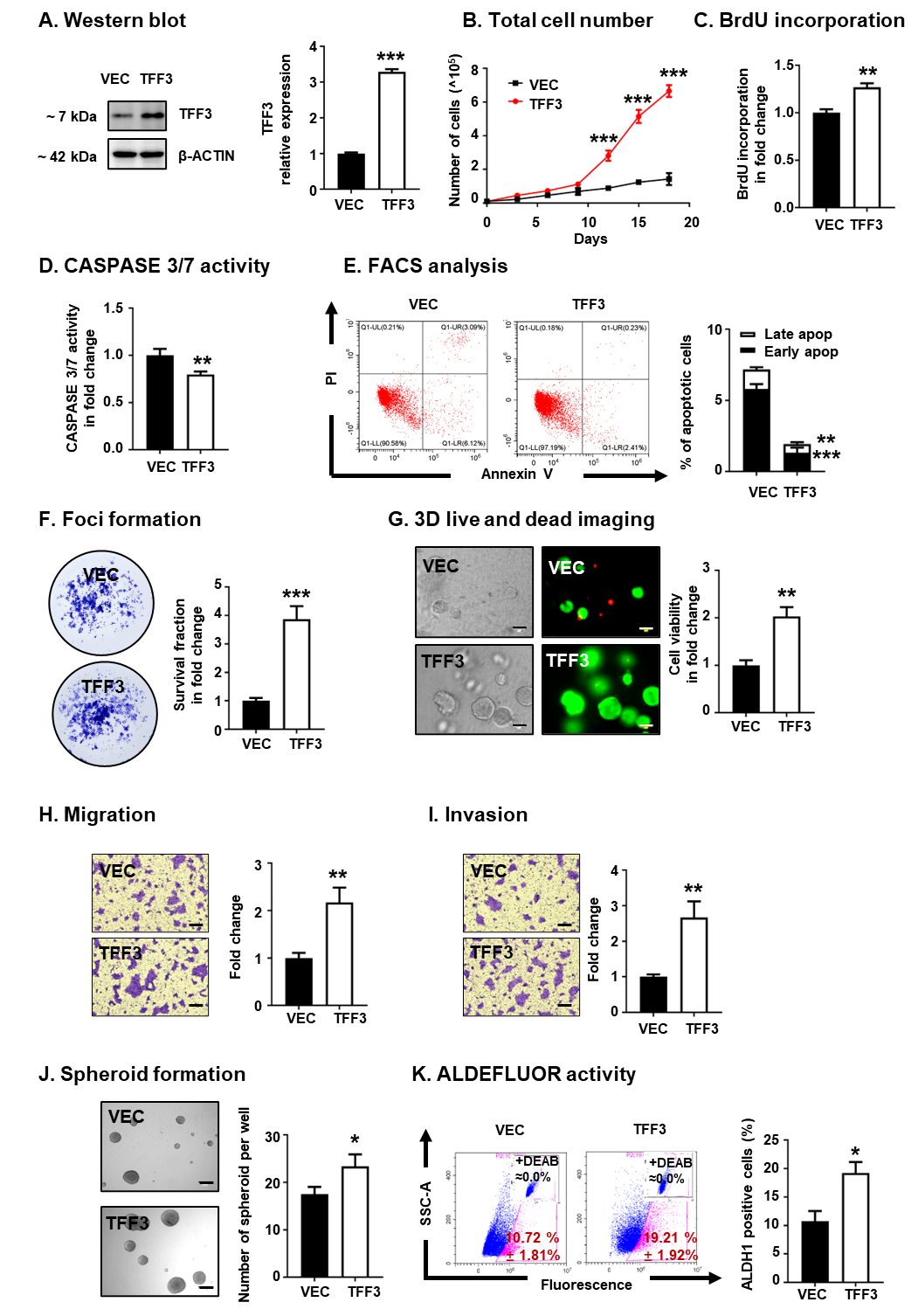


BT474 cells were stably transfected with *pIRESneo3-TFF3* (designated BT474-TFF3 (TFF3)) or *pIRESneo3-vector* (BT474-VEC (VEC)).

**A.** Western blot analysis was performed to determine the expression of TFF3 in BT474-VEC (VEC) and BT474-TFF3 (TFF3) cells. Densitometric analysis of western blots was conducted by using ImageJ software. Data are expressed as mean ± SD (*n* = 3). Statistical significance is indicated as **P* < 0.05, ***P* < 0.01, and ****P* < 0.001.

**B.** Total cell number counting. Data are expressed as mean ± SD (*n* = 3). Statistical significance is indicated as **P* < 0.05, ***P* < 0.01, and ****P* < 0.001.

**C.** BrdU incorporation assay after 12 hours of serum deprivation. Data are expressed as mean ± SD (*n* = 3). Statistical significance is indicated as **P* < 0.05, ***P* < 0.01, and ****P* < 0.001.

**D.** CASPASE 3/7 activity after 12 hours of serum deprivation. Data are expressed as mean ± SD (*n* = 3). Statistical significance is indicated as **P* < 0.05, ***P* < 0.01, and ****P* < 0.001.

**E.** Annexin-V/PI apoptotic cell death was determined after 12 hours of serum deprivation. The percentages of cells in early (Annexin-V-positive/PI negative) or late (Annexin-V-positive/PI-positive) apoptotic phase were analyzed and plotted by flow cytometry Data are expressed as mean ± SD (*n* = 3). Statistical significance is indicated as **P* < 0.05, ***P* < 0.01, and ****P* < 0.001.

**F.** Foci formation. BT474-TFF3 (TFF3) and BT474-VEC (VEC) cells were cultured in monolayer at low cell density for foci formation for 14 days. Foci were stained by crystal violet. Fold change of survival fraction was measured by eluting the crystal violet with methanol and detected absorbance at 595 nm. Data are expressed as mean ± SD (*n* = 3). Statistical significance is indicated as **P* < 0.05, ***P* < 0.01, and ****P* < 0.001.

**G.** Microscopic visualization of LIVE/DEAD^TM^ Cell Imaging kit-stained colonies after 12 days of cell culture in medium containing 2% FBS and 4% Matrigel. The bright-field images were on the left and the merged imaged of Live and Dead were on the right with red indicating dead colonies and green indicating live colonies. Scale bars, 100 μm. Fold change of cell viability of 3D colonies was determined by AlamarBlue. Data are expressed as mean ± SD (*n* = 3). Statistical significance is indicated as **P* < 0.05, ***P* < 0.01, and ****P* < 0.001.

**H.** Cell migration assay. Scale bar, 50 μm. Data are expressed as mean ± SD (*n* = 3). Statistical significance is indicated as **P* < 0.05, ***P* < 0.01, and ****P* < 0.001.

**I.** Cell invasion assay. Scale bar, 50 μm. Data are expressed as mean ± SD (*n* = 3). Statistical significance is indicated as **P* < 0.05, ***P* < 0.01, and ****P* < 0.001.

**J.** Spheroid formation assay. BT474-VEC (VEC)and BT474-TFF3 (TFF3) cells were seeded in ultralow attachment plates in and cultured in spheroid growth media for 12 days. Scale bar, 100 μm. The spheroids with diameters greater than 50 μm in each well were counted. Data are expressed as mean ± SD (*n* = 3). Statistical significance is indicated as **P* < 0.05, ***P* < 0.01, and ****P* < 0.001.

**K.** ALDEFLUOR activity after 12 hours of serum deprivation. The cells were then harvested and incubated with ALDEFLUOR substrate to define the ALDH1-positive population. DEAB was used to establish the baseline fluorescence. The percentage of ALDH1-positive cells (in the pink box) was plotted by flow cytometry. Data are expressed as mean ± SD (*n* = 3). Statistical significance is indicated as **P* < 0.05, ***P* < 0.01, and ****P* < 0.001.

**Supplementary information 3**


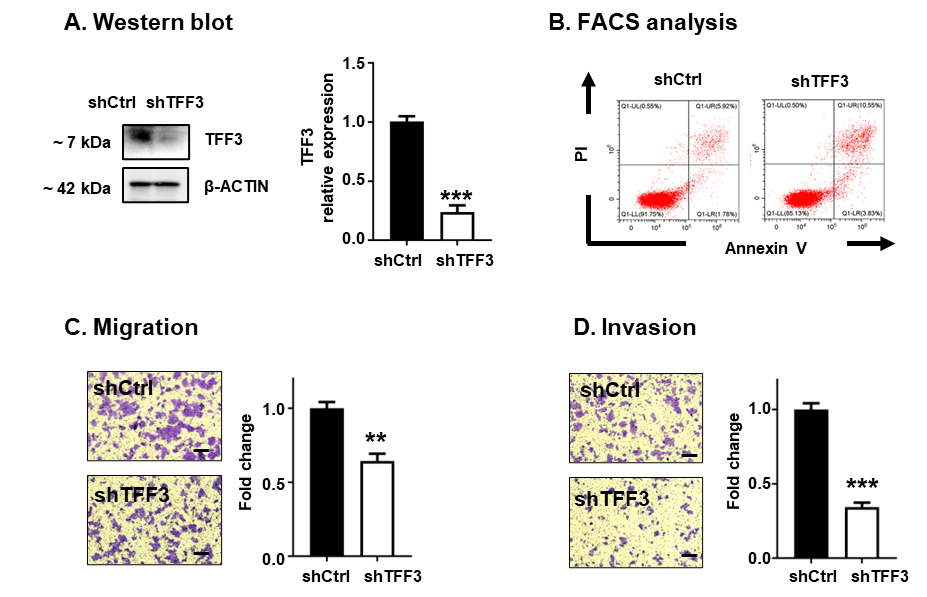


**A.** Western blot analysis was performed to determine the expression of TFF3 in MDA-MB-361-shCtrl (shCtrl) and MDA-MB-361-shTFF3 (shTFF3) cells. Densitometric analysis of western blots was conducted by using ImageJ software. Data are expressed as mean ± SD (*n* = 3). Statistical significance is indicated as **P* < 0.05, ***P* < 0.01, and ****P* < 0.001.

**B.** The percentage of early apoptotic (Annexin-V-positive/PI negative) and late apoptotic (Annexin-V-positive/PI-positive) cells was plotted by flow cytometry in MDA-MB-361-shCtrl (shCtrl) and MDA-MB-361-shTFF3 (shTFF3) cells.

**C.** Cell migration assay. Scale bar, 50 μm. Data are expressed as mean ± SD (*n* = 3). Statistical significance is indicated as **P* < 0.05, ***P* < 0.01, and ****P* < 0.001.

**D.** Cell invasion assay. Scale bar, 50 μm. Data are expressed as mean ± SD (*n* = 3). Statistical significance is indicated as **P* < 0.05, ***P* < 0.01, and ****P* < 0.001.

**Supplementary information 4**


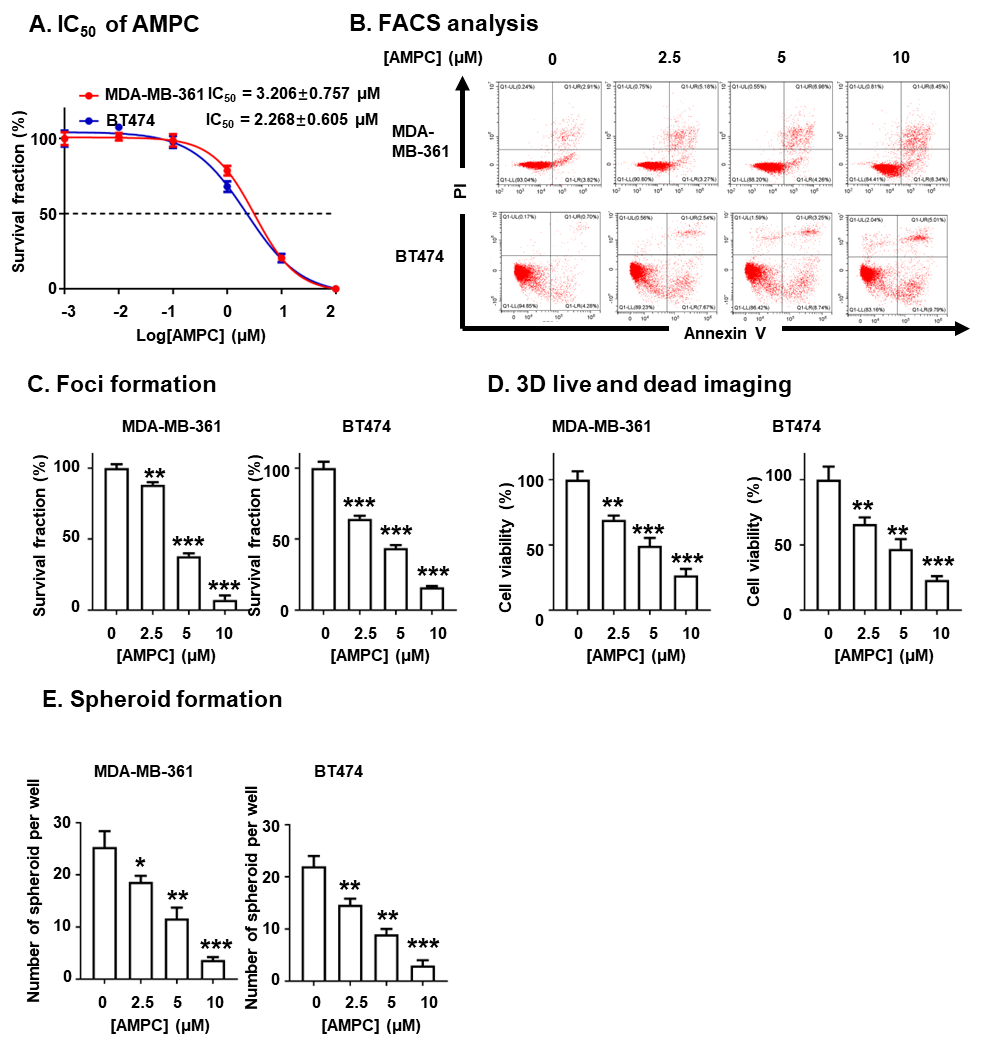


**A.** Effect of AMPC on the total cell number of ER+HER2+ MC cells was assessed using a total cell number counting assay after treatment with AMPC (0, 0.01, 0.1, 1, 10 or 100 µM) for 6 days. Inhibitory concentration 50% (IC_50_) values of AMPC for MDA-MB-361 and BT474 cells are presented as IC_50_ ± SD.

**B.** MDA-MB-361 and BT474 cells were treated with 0, 2.5, 5 or 10 µM AMPC for 2 days. The percentage of early apoptotic (Annexin-V positive/PI negative) and late apoptotic (Annexin-V positive/PI positive) cells was plotted by flow cytometry.

**C.** MDA-MB-361 and BT474 cells were treated with 0, 2.5, 5 or 10 µM AMPC in monolayer culture for 14 days. Foci were stained by crystal violet. Survival fraction was measured by eluting the crystal violet with methanol and determination of absorbance at 595 nm. Data are expressed as mean ± SD (*n* = 3). Statistical significance is indicated as **P* < 0.05, ***P* < 0.01, and ****P* < 0.001.

**D.** MDA-MB-361 and BT474 cells were treated with 0, 2.5, 5 or 10 µM AMPC in 3D-Matrigel culture for 12 days. Cell viability of 3D colonies was determined by AlamarBlue. Data are expressed as mean ± SD (*n* = 3). Statistical significance is indicated as **P* < 0.05, ***P* < 0.01, and ****P* < 0.001.

**E.** MDA-MB-361 and BT474 cells were cultured in ultralow attachment plates in spheroid growth media with 0, 2.5, 5 or 10 µM AMPC treatment for 12 days. The spheroids with diameters greater than 50 μm in each well were counted. Data are expressed as mean ± SD (*n* = 3). Statistical significance is indicated as **P* < 0.05, ***P* < 0.01, and ****P* < 0.001.

**Supplementary information 5**

| **Product Name** | **Pathway** | **Target** |
| --- | --- | --- |
| Dacomitinib (PF299804, PF299) | Protein Tyrosine Kinase | EGFR |
| Gefitinib (ZD1839) | Protein Tyrosine Kinase | EGFR |
| Nintedanib (BIBF 1120) | Protein Tyrosine Kinase | FGFR, PDGFR, VEGFR |
| OSI-930 | Protein Tyrosine Kinase | c-KIT, CSF-1R, VEGFR |
| PHA-665752 | Protein Tyrosine Kinase | c-MET |
| SU11274 | Protein Tyrosine Kinase | c-MET |
| Vatalanib (PTK787) 2HCl | Protein Tyrosine Kinase | VEGFR |
| BIBR 1532 | DNA Damage | Telomerase |
| Cyclophosphamide Monohydrate | DNA Damage | DNA alkylator |
| Doxorubicin (Adriamycin) HCl | DNA Damage | Topoisomerase |
| Iniparib (BSI-201) | DNA Damage | PARP |
| Rucaparib (AG-014699, PF-01367338) phosphate | DNA Damage | PARP |
| Aminoglutethimide | Endocrinology & Hormones | Aromatase |
| Bicalutamide | Endocrinology & Hormones | Androgen Receptor |
| GW9508 | Endocrinology & Hormones | GPR |
| Hydrocortisone | Endocrinology & Hormones | Glucocorticoid Receptor |
| Letrozole | Endocrinology & Hormones | Aromatase |
| AMG-900 | Cell Cycle | Aurora Kinase |
| Barasertib (AZD1152-HQPA) | Cell Cycle | Aurora Kinase |
| Y-27632 2HCl | Cell Cycle | Autophagy, ROCK |
| Combretastatin A4 | Cytoskeletal Signaling | Microtubule Associated |
| Luminespib (AUY-922, NVP-AUY922) | Cytoskeletal Signaling | HSP (e.g. HSP90) |
| Paclitaxel | Cytoskeletal Signaling | Autophagy, Microtubule Associated |
| Belinostat (PXD101) | Epigenetics | HDAC |
| BIX 01294 | Epigenetics | Histone Methyltransferase |
| Sirtinol | Epigenetics | Sirtuin |
| 2-Methoxyestradiol (2-MeOE2) | Angiogenesis | HIF |
| Saracatinib (AZD0530) | Angiogenesis | SRC |
| Obatoclax Mesylate (GX15-070) | Apoptosis | Autophagy, Bcl-2 |
| YM155 (Sepantronium Bromide) | Apoptosis | Survivin |
| Silmitasertib (CX-4945) | Metabolism | Casein Kinase |
| Tipifarnib | Metabolism | Transferase |
| CH5132799 | PI3K/Akt/mTOR | PI3K |
| Idelalisib (CAL-101, GS-1101) | PI3K/Akt/mTOR | PI3K |
| Febuxostat | Immunology & Inflammation | ROS |
| Doramapimod (BIRB 796) | MAPK | p38 MAPK |
| Maraviroc | Microbiology | CCR |
| Valproic acid sodium salt (Sodium valproate) | Neuronal Signaling | GABA Receptor, HDAC, Autophagy |
| QNZ (EVP4593) | NF-κB | NF-κB, TNF-alpha |
| Ixazomib Citrate (MLN9708) | Proteases | Proteasome |

The detailed information of the compounds showing synergistic effect with AMPC in both MDA-MB-361 and BT474 cells.

**Supplementary information 6**

**A. c-MET inhibitors and the structure**

| **c-MET inhibitors** | **Target** | **Structure** |
| --- | --- | --- |
| **PHA-665752 (P)** | c-MET, RON, FLK1 and c-ABL | 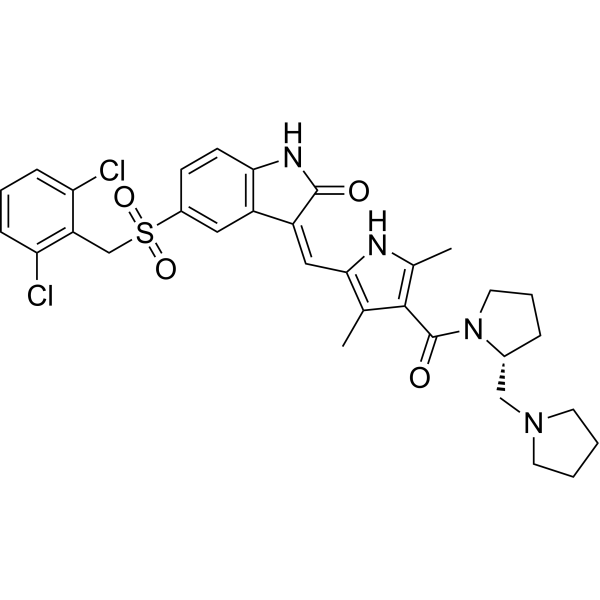 |
| **SU11274 (S)** | c-MET (a selective c-MET inhibitor) | 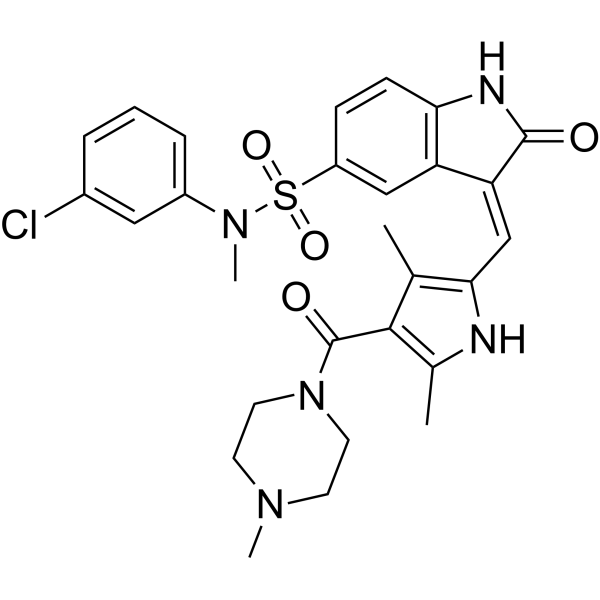 |
| **Cabozantinib (C)** | c-MET, VEGFR2, RET, c-KIT, AXL, FLT3 | 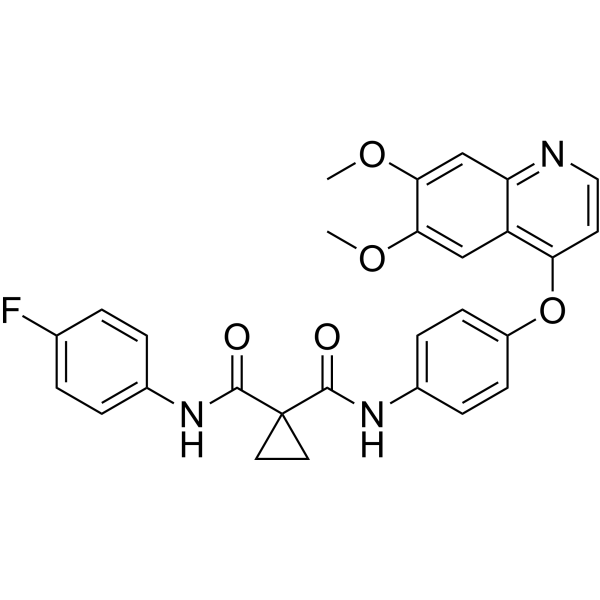 |

**B. IC_50_ of AMPC and c-MET inhibitors**

| **Cell line** | **c-METi** | **IC_50_ of c-METi (μM)** | **IC_50_ of AMPC+c-METi (μM)** |
| --- | --- | --- | --- |
| **MDA-MB-361** | C | 0.233±0.038 | 0.019±0.003 |
|  | S | 0.555±0.149 | 0.103±0.032 |
|  | P | 0.603±0.042 | 0.015±0.002 |
| **BT474** | C | 0.555±0.068 | 0.056±0.013 |
|  | S | 0.142±0.043 | 0.019±0.008 |
|  | P | 1.815±0.537 | 0.056±0.003 |

A. Targets and the structures of the c-METis.

B. The shift in IC_50_ of Cabozantinib (C), SU11274 (S), or PHA-665752 (P) in MDA-MB-361 and BT474 cells after co-treatment with AMPC (2.5 μM) was conducted with total cell number assay. Data are represented as means ± SD (*n*  =  3)

**Supplementary information 7**


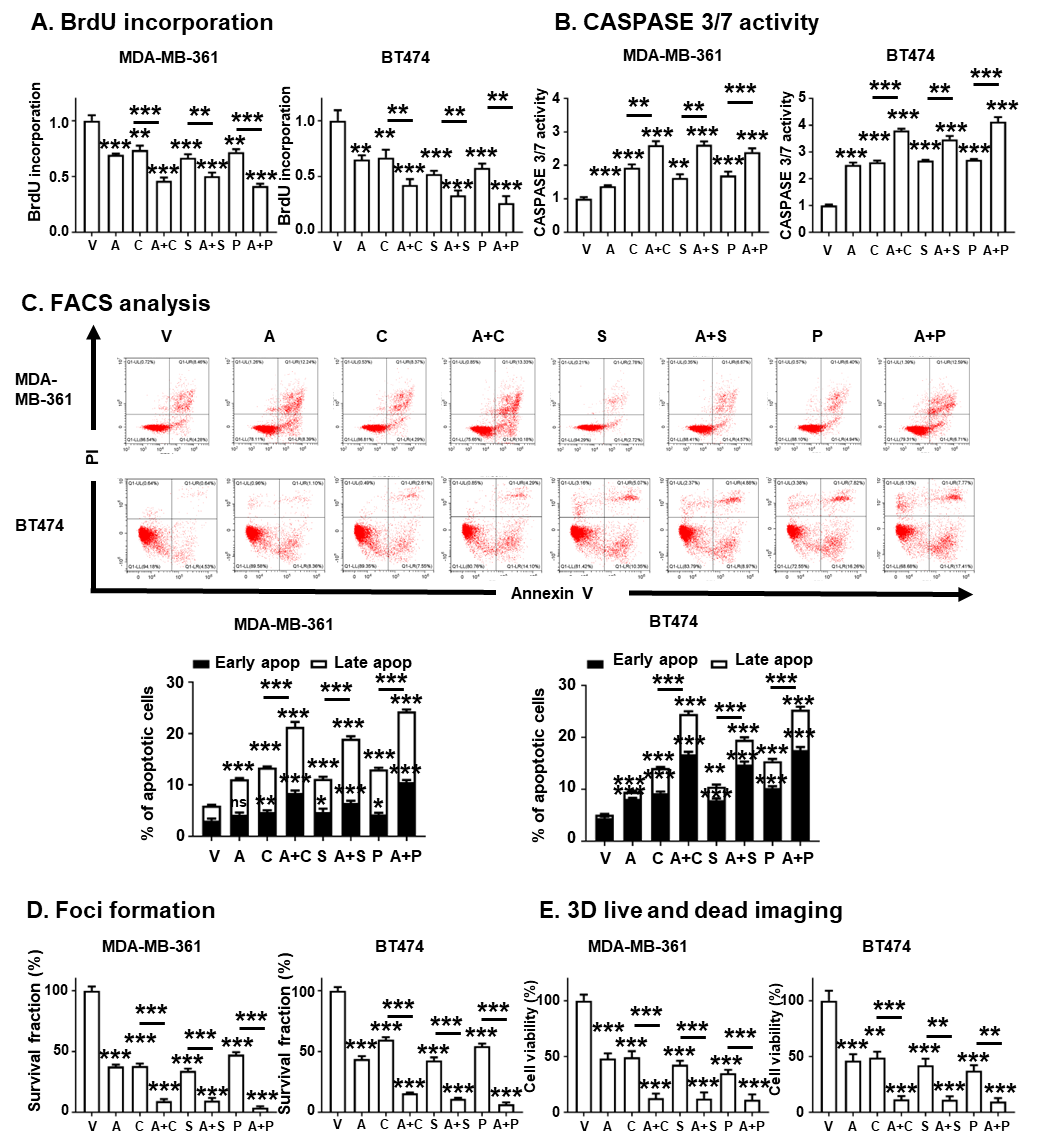


**A.** BrdU incorporation assays in MDA-MB-361 and BT474 cells were performed with the treatment of vehicle (V), 5 μM AMPC (A), 1 μM Cabozantinib (C), 1 μM SU11274 (S), 2 μM PHA-665752 (P) or the combination for 3 days. Data are expressed as mean ± SD (*n* = 3). Statistical significance is indicated as **P* < 0.05, ***P* < 0.01, and ****P* < 0.001.

**B.** CASPASE 3/7 activities were determined after 3 days of vehicle (V), 5 μM AMPC (A), 1 μM Cabozantinib (C), 1 μM SU11274 (S), 2 μM PHA-665752 (P) or the combination treatment. Data are expressed as mean ± SD (*n* = 3). Statistical significance is indicated as **P* < 0.05, ***P* < 0.01, and ****P* < 0.001.

**C.** Apoptotic cell death of MDA-MB-361 and BT474 cells was determined after 2 days of vehicle (V), 5 μM AMPC (A), 1 μM Cabozantinib (C), 1 μM SU11274 (S), 2 μM PHA-665752 (P) or the combination treatment using Annexin-V/PI. The percentage of early apoptotic (Annexin-V positive/PI negative) and late apoptotic (Annexin-V positive/PI positive) cells was plotted by flow cytometry and analyzed. Data are expressed as mean ± SD (*n* = 3). Statistical significance is indicated as **P* < 0.05, ***P* < 0.01, and ****P* < 0.001.

**D.** Foci formation. MDA-MB-361 and BT474 cells were treated with vehicle (V), 5 μM AMPC (A), 1 μM Cabozantinib (C), 1 μM SU11274 (S), 2 μM PHA-665752 (P) or the combination for foci formation for 14 days. Foci were stained by crystal violet. The survival fraction was measured by eluting the crystal violet with methanol and determination of absorbance at 595 nm. Data are expressed as mean ± SD (*n* = 3). Statistical significance is indicated as **P* < 0.05, ***P* < 0.01, and ****P* < 0.001.

**E.** MDA-MB-361 and BT474 cells were treated with vehicle (V), 5 μM AMPC (A), 1 μM Cabozantinib (C), 1 μM SU11274 (S), 2 μM PHA-665752 (P) or the combination in 3D-Matrigel culture for 12 days after 3 days of pre-culture. Cell viability of 3D colonies was determined by AlamarBlue. Data are expressed as mean ± SD (*n* = 3). Statistical significance is indicated as **P* < 0.05, ***P* < 0.01, and ****P* < 0.001.

**Supplementary information 8**


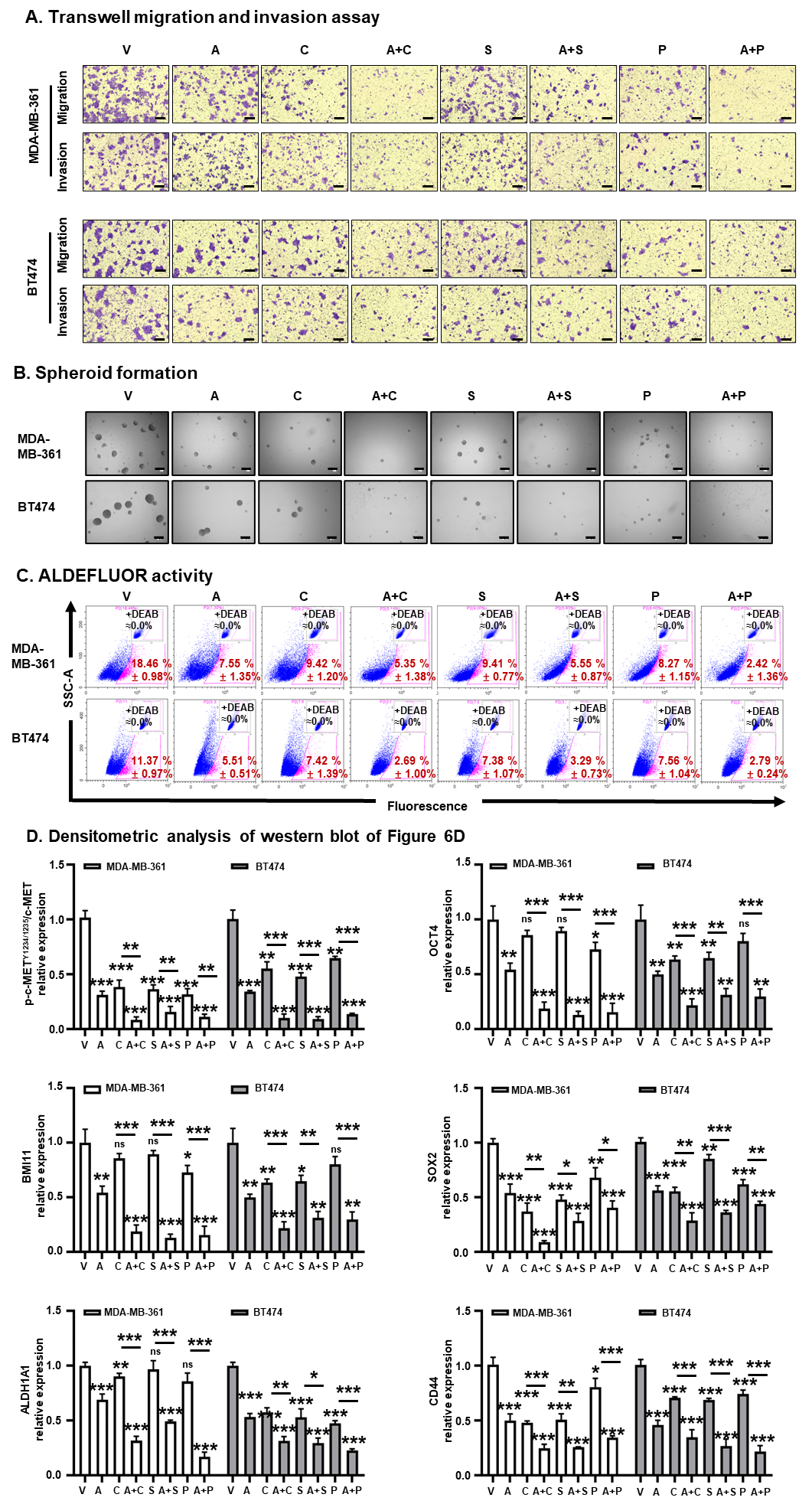


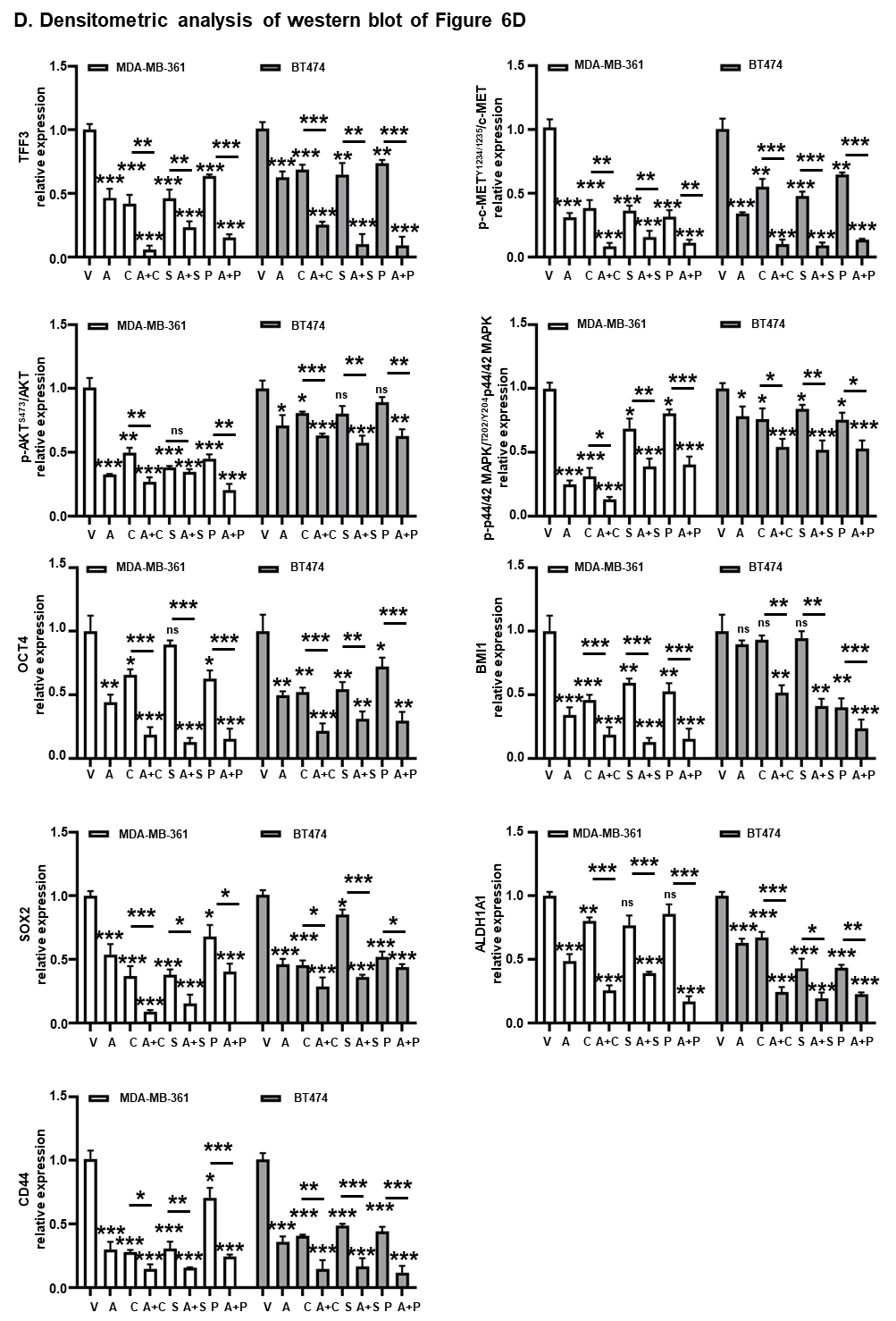
**A.** Cell migration and invasion assay were performed on MDA-MB-361 and BT474 cells treated with vehicle (V), 5 μM AMPC (A), 1 μM Cabozantinib (C), 1 μM SU11274 (S), 2 μM PHA-665752 (P) or the combination. Scale bar, 50 μm.

**B.** MDA-MB-361 and BT474 cells were seeded in ultralow attachment plates and cultured in spheroid growth media, and treated with vehicle (V), 5 μM AMPC (A), 1 μM Cabozantinib (C), 1 μM SU11274 (S), 2 μM PHA-665752 (P) or the combination for 12 days. Scale bar, 50 μm.

**C.** ALDEFLUOR assay analysis was measured on MDA-MB-361 and BT474 cells with the treatment of vehicle (V), 5 μM AMPC (A), 1 μM Cabozantinib (C), 1 μM SU11274 (S), 2 μM PHA-665752 (P) and the combination for 3 days. The percentage of ALDH1-positive cells (in the pink box) was plotted by flow cytometry. Data are expressed as mean ± SD (*n* = 3).

**D.** Densitometric analysis of western blots in Figure 6D was conducted by using ImageJ software. Data are expressed as mean ± SD (*n* = 3). Statistical significance is indicated as **P* < 0.05, ***P* < 0.01, and ****P* < 0.001.

**Supplementary information 9**


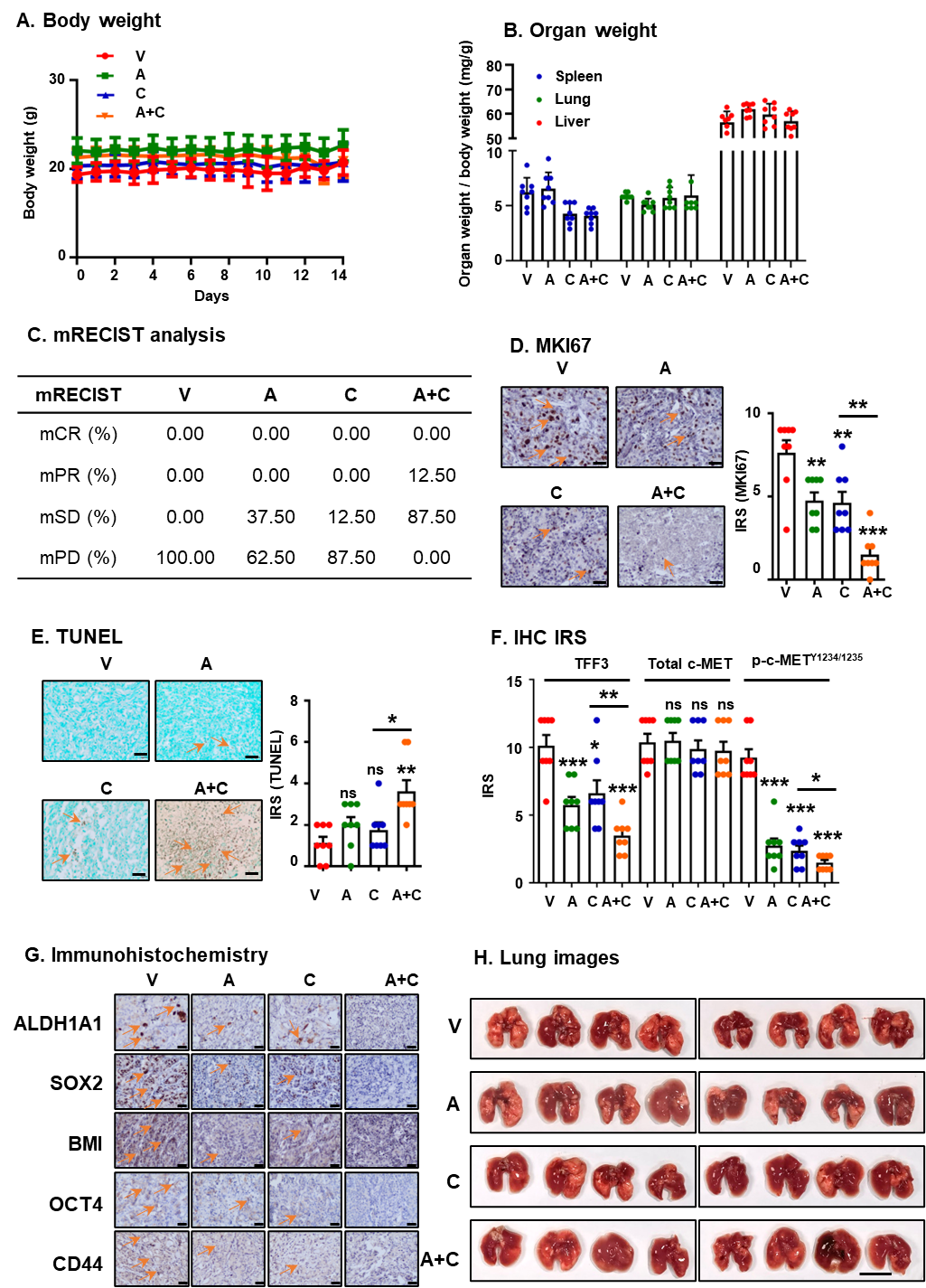


**A.** Body weights of mice were recorded during the treatment period. Data are expressed as mean ± SD (*n* = 8). Statistical significance is indicated as **P* < 0.05, ***P* < 0.01, and ****P* < 0.001.

**B.** Weights of resected organs was determined at the termination of the experiments. Data are expressed as mean ± SD (*n* = 8). Statistical significance is indicated as **P* < 0.05, ***P* < 0.01, and ****P* < 0.001.

**C.** The modified Response Evaluation Criteria in Solid Tumors (mRECIST) assessment was performed. CR: disappearance of any intertumoral arterial enhancement in all target lesions, PR: at least a 30% decrease in the sum of diameters of viable (enhancement in the arterial phase) target lesions, taking as reference the baseline sum of the diameters of target lesions, SD: Any cases that do not qualify for either partial response or progressive disease, PD: an increase of at least 20% in the sum of the diameters of viable (enhancing) target lesions, taking as reference the smallest sum of the diameters of viable (enhancing) target lesions recorded since treatment started.

**D.** IHC and IRS analysis on MKI67 were conducted in the indicated xenografts. Scale bar, 20 μm. Data are expressed as mean ± SD (*n* = 8). Statistical significance is indicated as **P* < 0.05, ***P* < 0.01, and ****P* < 0.001.

**E.** TUNEL and IRS analysis were performed in the indicated xenografts. Scale bar, 20 μm. Data are expressed as mean ± SD (*n* = 8). Statistical significance is indicated as **P* < 0.05, ***P* < 0.01, and ****P* < 0.001.

**F.** IRS score of TFF3, c-MET and c-MET phosphorylation at Y1234/1235 was assessed. Data are expressed as mean ± SD (*n* = 8). Statistical significance is indicated as **P* < 0.05, ***P* < 0.01, and ****P* < 0.001.

**G.** IHC staining for CSC markers in the indicated xenografts. Scale bar, 20 μm.

**H.** Photographs of lungs of mice after vehicle (V), AMPC (A), Cabozantinib (C) and AMPC + Cabozantinib (A+C) treatment. Scale bar, 1 cm.


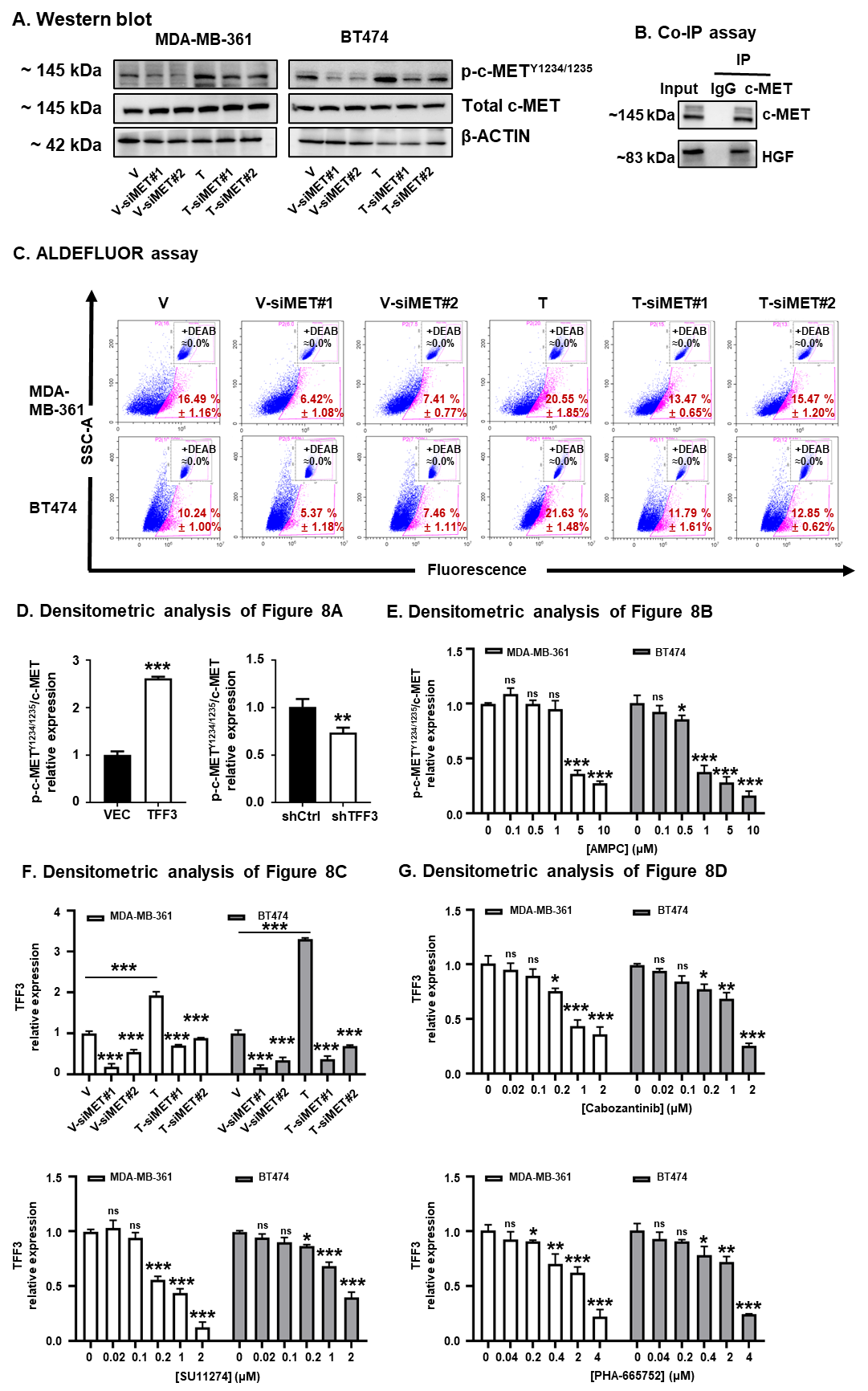
**Supplementary information 10**


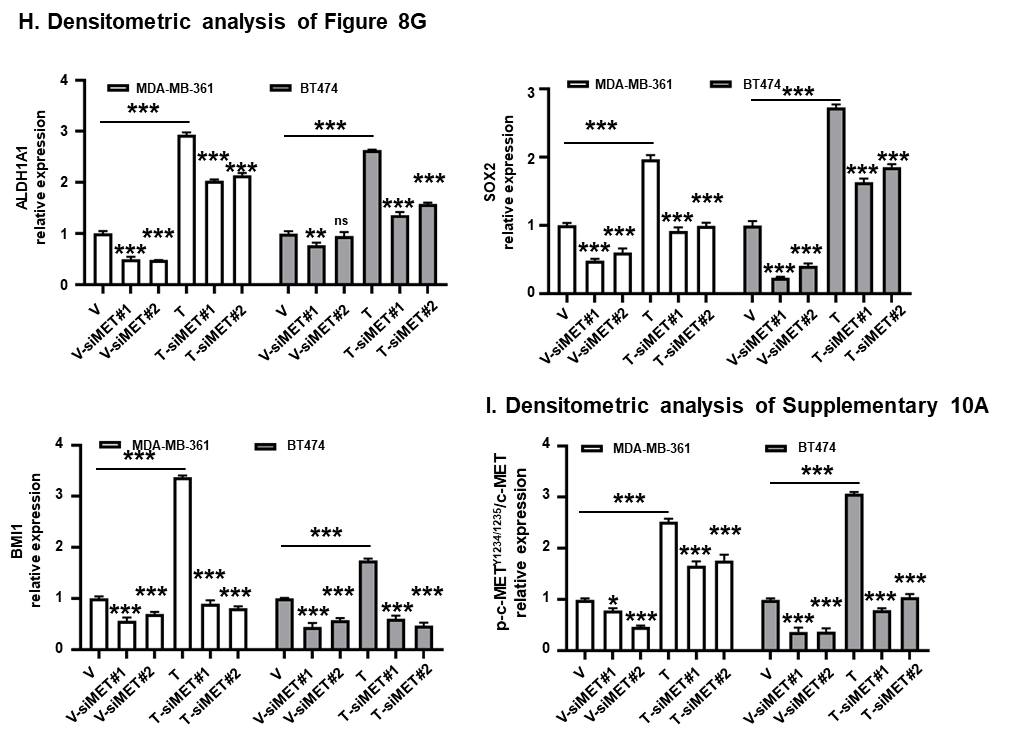


**A.** Western blot analysis was performed to assess the level of c-MET and c-MET phosphorylation at Y1234/1235 in MDA-MB-361 and BT474 cells transfected with scrambled siRNA, siMET#1 or siMET#2 plasmid. β-ACTIN was used as input control. The sizes of detected protein blots in kDa were shown on the left.

**B.** A potential c-MET/TFF3 interaction in MDA-MB-361 cells was investigated by immunoprecipitation (IP) and immunoblotting. HGF was used as the positive control.

**C.** ALDEFLUOR assay analysis of MDA-MB-361 and BT474 cells transfected with scrambled siRNA, siMET#1 and siMET#2. The percentage of ALDH1-positive cells (in the pink box) was plotted by flow cytometry. Data were expressed as mean ± SD (*n*=3). Statistical significance was indicated as **P* < 0.05, ***P* < 0.01, and ****P* < 0.001.

**D.** Densitometric analysis of western blots in Figure 8A was conducted by using ImageJ software. Data were expressed as mean ± SD (*n*=3). Statistical significance was indicated as **P* < 0.05, ***P* < 0.01, and ****P* < 0.001.

**E.** Densitometric analysis of western blots in Figure 8B was conducted by using ImageJ software. Data were expressed as mean ± SD (*n*=3). Statistical significance was indicated as **P* < 0.05, ***P* < 0.01, and ****P* < 0.001.

**F.** Densitometric analysis of western blots in Figure 8C was conducted by using ImageJ software. Data were expressed as mean ± SD (*n*=3). Statistical significance was indicated as **P* < 0.05, ***P* < 0.01, and ****P* < 0.001.

**G.** Densitometric analysis of western blots in Figure 8D was conducted by using ImageJ software. Data were expressed as mean ± SD (*n*=3). Statistical significance was indicated as **P* < 0.05, ***P* < 0.01, and ****P* < 0.001.

**H.** Densitometric analysis of western blots in Figure 8G was conducted by using ImageJ software. Data were expressed as mean ± SD (*n*=3). Statistical significance was indicated as **P* < 0.05, ***P* < 0.01, and ****P* < 0.001.

**I.** Densitometric analysis of western blots in Supplementary information 10A was conducted by using ImageJ software. Data were expressed as mean ± SD (*n*=3). Statistical significance was indicated as **P* < 0.05, ***P* < 0.01, and ****P* < 0.001.

**Supplementary information 11**

| **Antibody** | **Vendor** | **Catalog** | **Application** | **Dilution** |
| --- | --- | --- | --- | --- |
| TFF3 | Abcam | ab108599 | WB/ IHC/ IF | 1:1000/1:200/1:200 |
| MKI67 | Abcam | ab16667 | IHC | 1:500 |
| β-ACTIN | Santa Cruz | sc-47778 | WB | 1:1000 |
| Total MET | CST | 8198S | WB/ IHC | 1:1000/1:100 |
| Phospho-MET Y1234/1235 | CST | 3077S | WB/ IHC | 1:1000/1:300 |
| Total AKT | Abcam | ab8805 | WB | 1:1000 |
| Phospho-AKT S473 | Abcam | ab66138 | WB | 1:1000 |
| Total p44/42 MAPK | CST | 4695S | WB | 1:1000 |
| Phospho- p44/42 MAPK T202/Y204 | CST | 4370S | WB | 1:1000 |
| OCT4 | Abcam | ab181557 | WB/ IHC | 1:1000/1:300 |
| BMI1 | CST | 6964 | WB/ IHC | 1:1000/1:250 |
| ALDH1A1 | CST | 36671 | WB/ IHC | 1:1000/1:250 |
| CD44 | Abcam | ab157107 | WB/ IHC | 1:1000/1:250 |
| SOX2 | Abcam | ab97959 | WB/ IHC | 1:1000/1:100 |
| hHPRT | Invitrogen | MA5-15274 | IHC | 1:200 |
| Rabit IgG | CST | 7074 | WB | 1:3000 |
| Mouse IgG | CST | 7076 | WB | 1:3000 |
