# Supplementary figures and images for: Inhibition of TFF3 synergizes with c-MET inhibitors to decrease the CSC-like phenotype and metastatic burden in ER+HER2+ mammary carcinoma
Source: Cell Death Dis. 2025 Feb 7;16(1):76. doi: 10.1038/s41419-025-07387-5 (PMC11806102; doi:10.1038/s41419-025-07387-5)

**Supplementary information for uncropped western blot images.**


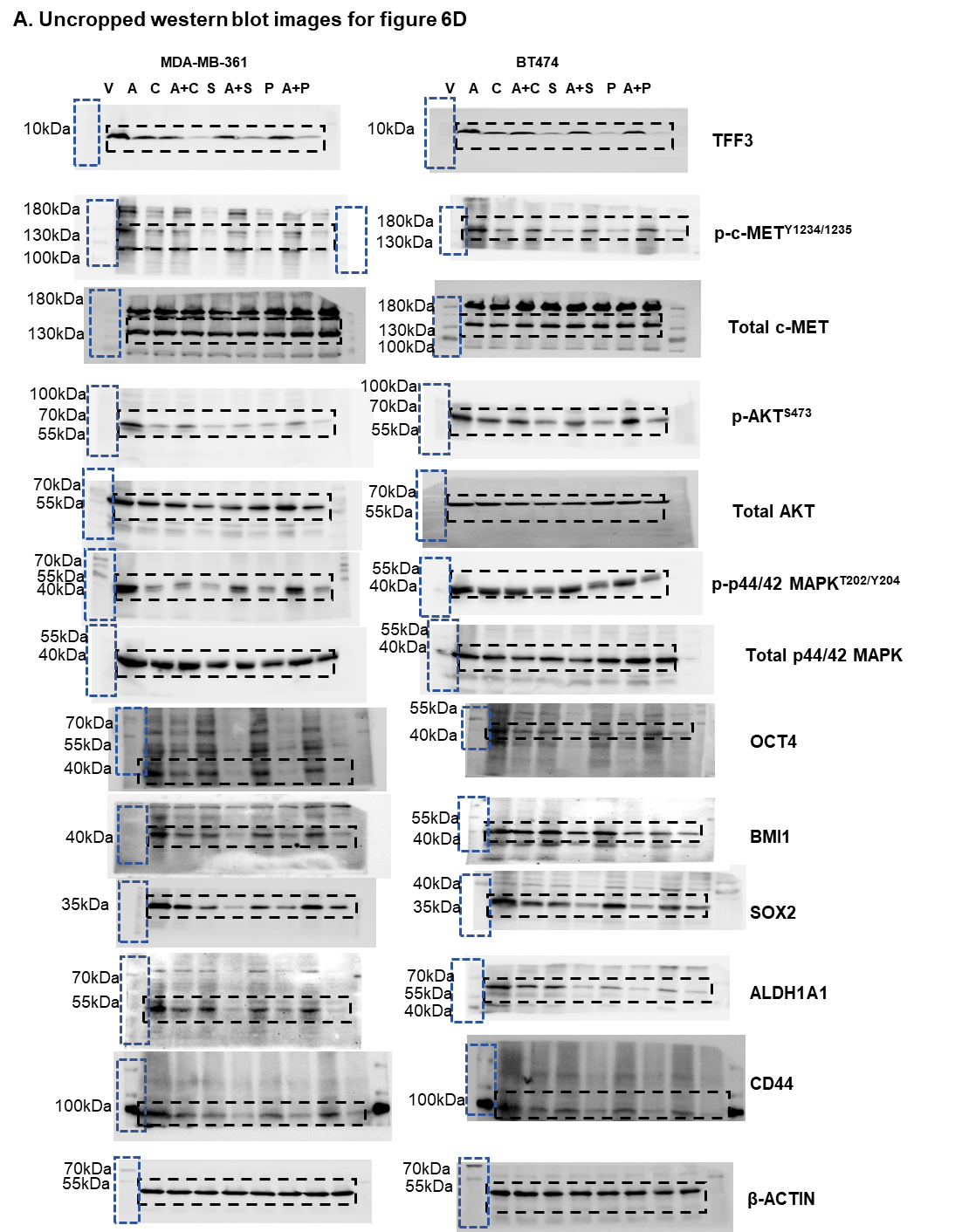


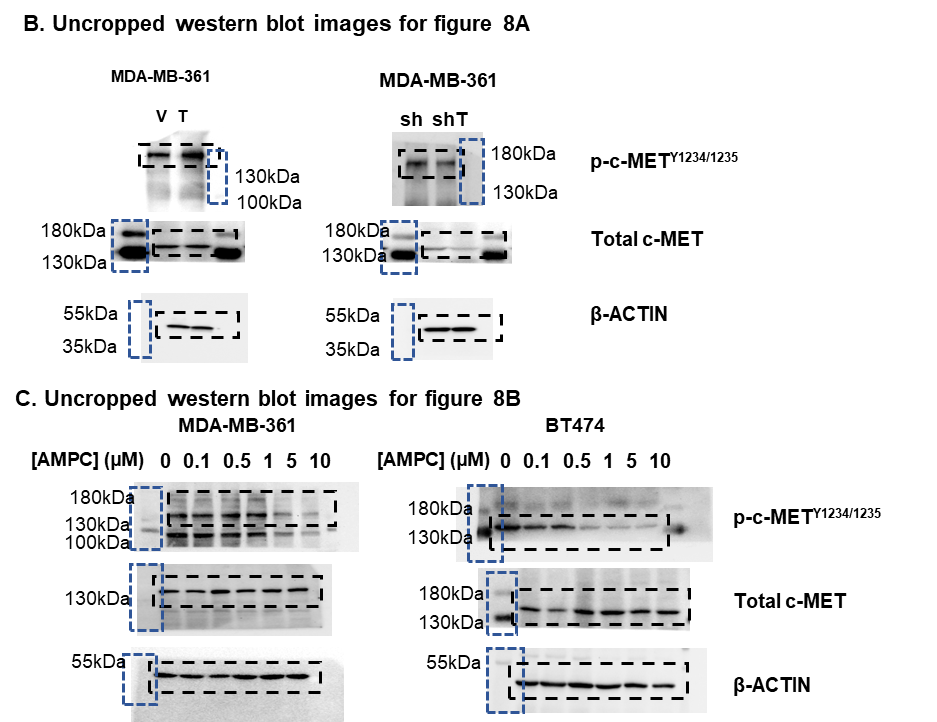


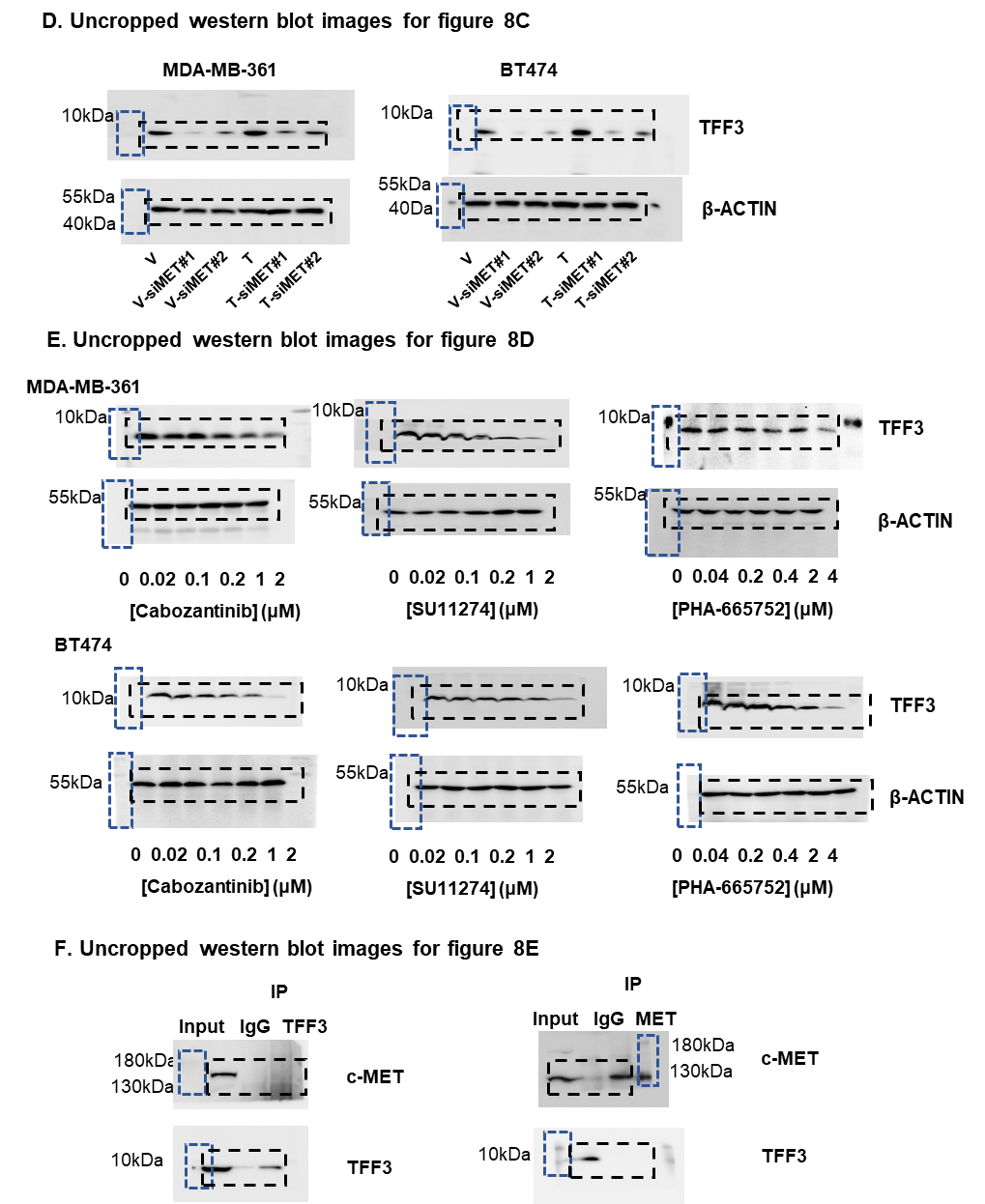


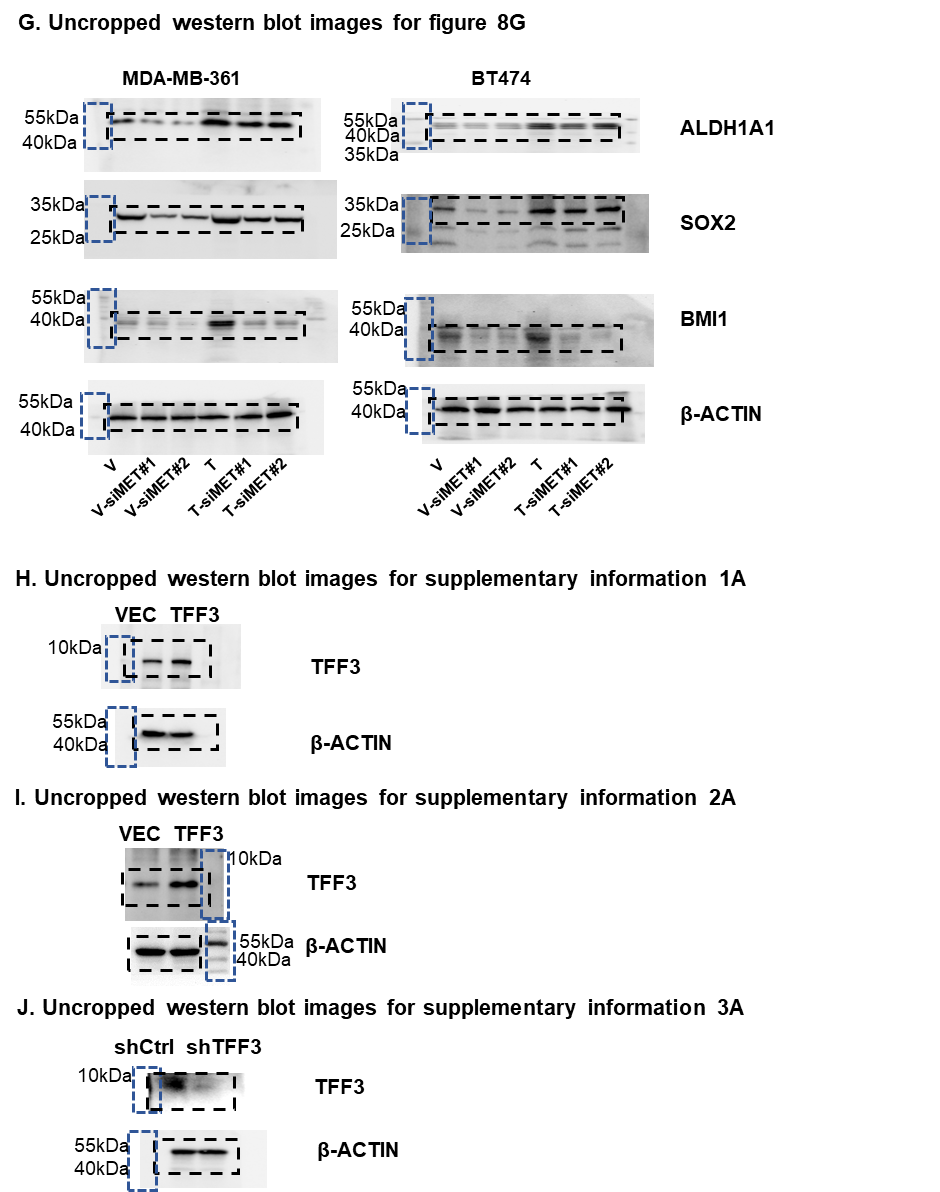


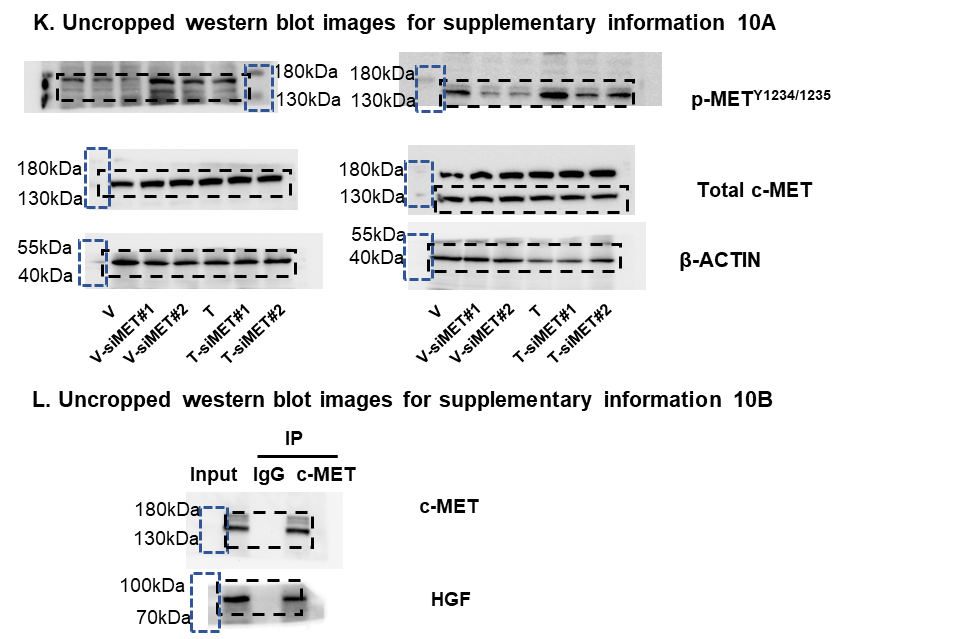

Supplement: Supplementary file 2 — Supplementary information for uncropped western blot images [file 41419_2025_7387_MOESM2_ESM.docx]
